# Comparative Phylogenomics of Pathogenic and Non-Pathogenic Mycobacterium

**DOI:** 10.1371/journal.pone.0071248

**Published:** 2013-08-28

**Authors:** Arun N. Prasanna, Sarika Mehra

**Affiliations:** Department of Chemical Engineering, Indian Institute of Technology Bombay, Powai, Mumbai, India; Beijing Institute of Genomics, China

## Abstract

*Mycobacterium* species are the source of a variety of infectious diseases in a range of hosts. Genome based methods are used to understand the adaptation of each pathogenic species to its unique niche. In this work, we report the comparison of pathogenic and non-pathogenic *Mycobacterium* genomes. Phylogenetic trees were constructed using sequence of core orthologs, gene content and gene order. It is found that the genome based methods can better resolve the inter-species evolutionary distances compared to the conventional 16S based tree. Phylogeny based on gene order highlights distinct evolutionary characteristics as compared to the methods based on sequence, as illustrated by the shift in the relative position of *M. abscessus*. This difference in gene order among the *Mycobacterium* species is further investigated using a detailed synteny analysis. It is found that while rearrangements between some *Mycobacterium* genomes are local within synteny blocks, few possess global rearrangements across the genomes. The study illustrates how a combination of different genome based methods is essential to build a robust phylogenetic relationship between closely related organisms.

## Introduction

*Mycobacterium* genus of pathogenic species causes a variety of infectious diseases in hosts ranging from humans to fish. Together, infections caused by this group of species are responsible for millions of death annually among different life forms. The well-known infections, tuberculosis and leprosy are caused by *M. tuberculosis* and *M. leprae* respectively [1]. In addition, *M. ulcerans* is responsible for skin ulcers in humans and *M. avium* leads to opportunistic infections, such as in immune HIV positive compromised patients [2]. While many genes contributing to pathogenesis have been identified [3], [4], understanding the mechanisms of pathogenesis is an active area of research.

In addition to these pathogenic species, the family also consists of many non-pathogenic species such as *M. smegmatis*, *M. gilvum* and *M. vanbaalenii*. These non-pathogenic relatives, particularly, *M. smegmatis* have proved as useful hosts to express and study genes from pathogenic species. Comparative genomics of pathogenic and non-pathogenic species can help identify disease-related genes and vaccine candidates, and shed light on how each mycobacteria survives in its exclusive niche. A comparative study between various species also provides insight into their evolutionary relationship.

Analysis of vaccine and virulent strains of *M. tuberculosis* complex has identified regions that are found to be deleted in the former. Also, the RD1 region has been lost from many strains including *M. microti* and others. Another study found that indels are more frequent in *M. tuberculosis* than SNPs [5]. Further, large-scale gene inactivation and shrinkage in the genome of *M. leprae* was revealed on its comparison with *M. tuberculosis* [6]. Horizontal gene transfer has also played an important role in evolution of various *Mycobacterium* species [7], [8].

A direct comparison of minimal sets of ordered clones from BAC libraries representing the complete genome of *M. tuberculosis* H37Rv with the avirulent BCG strain revealed two major rearrangements in the BCG genome due to tandem duplication events [9]. A recent study of *Mycobacterium* and related species also identified specific functional categories such as lipid metabolism that are enriched in *M. tuberculosis* complex genomes [10].

In addition to whole genome comparisons, many studies have focussed on important classes of gene families such as sigma factors [11], proteases [12] and dormancy regulon genes [13]. Comparison of metabolic pathways in *M. tuberculosis*, *M. bovis*, *M. leprae* and *M. avium* showed major differences in cell wall and PE/PPE related genes [14]. Comparison of mycolic acid pathways across mycobacterial genomes has also been studied [15], [16].

In this work, comparative genome analysis is performed on 10 *Mycobacterium* genomes comprising of both pathogens and non-pathogens. Employing a phylogenomics approach, the study aims to compare the species in terms of sequence conservation, number of orthologs, genome organization and synteny. Orthologs are computed between all pairs of mycobacterial genomes. This set is used to further identify genes conserved across all mycobacteria considered in this study. Phylogenetic trees are constructed based on individual gene sequences and that of conserved genes. Order of core orthologs on the genome is also used to determine phylogenetic relationship between different species. A detailed gene synteny analysis is presented and genes specific to the pathogenic and non-pathogenic group are identified.

## Methods

### Identification of Homologs

Genome information for all the mycobacteria was downloaded from NCBI (ftp://ftp.ncbi.nih.gov/genomes/Bacteria/). Sequence alignment was performed using standalone version of BLAST program [17]. A bi-directional BLAST was performed between all gene/protein sequences for every pair of mycobacteria genomes. To perform the bidirectional BLAST, a local database was created from one genome. The second genome was queried against this database. The BLAST was repeated by interchanging the database and query genomes. Search for hits was performed for both nucleotide and protein sequences at a stringent E-value of 10^−6^. For every gene, the BLAST hit was considered significant if the overall percentage identity was greater than 50% and the alignment length greater than 50% of the reference gene in the database. Two genes are called homologs only if both the nucleotide and protein sequences satisfy the above criteria.

Genes were classified into functional categories based on the role and sub role classification available in CMR/TIGR [18]. The classification was downloaded for all genomes except for *M. ulcerans and M. marinum*. The role and sub-role assignment was not available for these organisms.

### Identification of Orthologs

To identify orthologs from the list of homologs, the best hit in terms of identity is identified among the similar pair of genes. For every pair of homologs, an average identity is calculated. Two homologs are called an ortholog if they are the best hit of each other. For example, gene g_1_ and g_2_ from genomes A and B respectively are orthologs if g_1_ has the highest identity with g_2_ among all the homologs of g_1_ in genome B and vice-versa.

Core orthologs were defined as a set of genes such that corresponding to every member, an ortholog from all the other genomes is also present in the set. To identify this set of core orthologs, an intersection of orthologs, O_i_ where i = 1 to n, was constructed. O_1_, defined as [A∩B…N] is the subset obtained by intersecting genes in organism A with its orthologs in all other organisms. Similarly, O_2_ is the subset obtained by intersecting genes of organism B with its orthologs in all other organisms. Core Orthologs (C.O) are defined as genes that are present in all the sets O_1_ to O_n_, i.e, C.O = [O_1_∩O_2_∩…O_n_].

### Phylogenetic Analysis

Phylogenetic analysis was performed in PHYLIP [19] using both distance based methods and maximum likelihood algorithm. For trees based on 16S and dnaN nucleotide sequences, distance was computed using the Jukes-Cantor model [20] available in the dnadist program. Similarly, for trees based on protein sequences the Jones-Taylor-Thornton (JTT) model [21] was used to compute distances between two protein sequences. For a given distance matrix, trees were constructed using Fitch-Margoliash (FM), Minimum Evolution, Neighbour-Joining [8] and UPGMA methods. Consensus trees were obtained based on majority rule (extended) using the program CONSENSE in PHYLIP [22]. Trees were visualized in itol [23]. To test the reliability of the tree branches, a bootstrap analysis with 1000 replicates was performed.

To study the evolutionary relationship between the various mycobacteria, protein sequences of the genes conserved across all mycobacteria was used. Protein sequences of the core orthologs were concatenated, and the resulting 10 sequences each representing one organism, were subjected to multiple sequence alignment on the MAFFT web server [24]. The BLOSUM62 matrix was used. Large gap segments in the alignment file was trimmed using trimal [25]. Both the programs were run with default settings. Phylogenetic analysis was performed in MEGA [26] using neighbour-joining, minimum evolution and UPGMA algorithms. Bootstrap was performed for each of the trees with 1000 replicates.

### Gene content analysis

Percentage shared gene content [27] is defined as the ratio of the number of orthologs shared between two genomes to the number of genes in the reference genome, where the reference genome can be either the smaller or the larger genome. Both measures have been used in the paper. A distance matrix was thus computed where distance is defined as 1- % gene content. Phylogenetic trees were constructed as described in the previous section.

### Genome Rearrangement analysis

To identify phylogenetic relationship between organisms based on gene order, the position of core orthologs on the chromosome was determined based on their start position. Each genome is represented as a signed permutation, 1, …, n, where a positive sign indicates coding strand and vice-versa. The order of genes on any one organism is considered as a reference. The process was repeated by considering each of the organisms as reference. Gene order distance between two genomes is defined as the number of reversals in one genome that would result in the same order of genes as that of the second genome. The reversal distance was computed in GRIMM [28] assuming all genomes to be unichromosomal and circular. The distance matrix was used to compute phylogenetic trees as described earlier. To obtain statistical support for the tree branches, a jackknife resampling approach [29] was followed. 40% of the genes were removed randomly from the initial core orthologs to obtain 50 jackknife sets. Tree was constructed from each of these sets and combined in CONSENSE as described earlier. The support values computed as percentage were assigned to the branches of the original gene order tree.

### Identification of conserved gene order

Orthologous genes were divided into units based on their physical location on the genome. A unit/block was defined as a chromosomal segment of a minimum pre-defined number of orthologous genes that share the same relative ordering on the chromosome of two mycobacterial genomes. The gene order and proximity should be conserved in both the genomes. Between two such conserved genes, a maximum of 5 non-homologous/inter-leaving genes could be present. i.e. the conserved gene order can be interrupted by a maximum of 5 genes.

## Results

### General Features of *Mycobacterium* Genomes

The genus *Mycobacterium* comprises of rod-shaped gram-positive bacteria with characteristic acid-fast structures. For the comparative genomics study presented in this work, 6 pathogenic and 4 non-pathogenic species were selected from among the completely sequenced *Mycobacterium* genomes. The genome of these 10 species (see Table 1) varies from 3.2 MB to 7 MB with an average GC content of 64–69%. With the largest genome size, *Mycobacterium smegmatis* has the highest number of genes and proteins. The smallest genome is that of *Mycobacterium leprae* with only 2770 genes, a large fraction of which is pseudogenes resulting in only 1605 proteins. The average GC content of *M. leprae* is also relatively lower at only 58%.

**Table 1:** Genome characteristics of mycobacterial pathogens and non-pathogens considered in this study.

ATTRIBUTES	*M. tuberculosis* H37Rv	*M. abscessus* ATCC 19977	*M. avium* 104	*M. leprae* TN	*M. ulcerans* Agy99	*M. marinum*	*M. smegmatis* mc2 155	*M. sp*. KMS	*M. gilvum* PYR-GCK	*M. vanbaalenii* PYR-1
**Genome size (MB)**	4.4	5.1	5.5	3.27	5.63	6.64	6.99	5.74	5.62	6.49
**# genes**	4062	4991	5313	2770	4957	5541	6938	5551	5327	6136
**# proteins**	4003	4941	5120	1605	4160	5423	6717	5460	5241	5979
**% GC content**	65.6	64.1	69	57.8	65.5	65.7	67.4	68.4	67.9	67.8
**Pathogenic**	YES	YES	YES	YES	YES	YES	NO	NO	NO	NO
**Disease caused to human**	TB	Skin infections	Pulmonary disease	Leprosy	Buruli ulcer	Lymphan-gitis	STL** (rare)	N.A	N.A	N.A
**Doubling time**	∼24 h	4–5 h	3.4 h–7 d	12–14 d	36 h	6–11 h	∼2–3 h	7–8 h	N.A	N.A
**Gram stain**	+	+	+	+	+	+	+	+	+	+
**Shape**	Bacilli	Bacilli	Bacilli	Bacilli	Bacilli	Bacilli	Bacilli	Bacilli	Bacilli	Bacilli
**Pseudo genes**	8	0	143	1115	747	67	167	34	32	99
**Structural RNA**	50	50	50	50	50	51	54	57	55	58
**Plasmids**	NIL	1	NIL	NIL	1	1	NIL	2	3	NIL
**Unique gene families**	3617	4614	4521	1581	3635	4738	6157	5034	4790	5418

** STL stands for Soft tissue Lesions.

The doubling time of the 10 species is inversely proportional to the size of their genome. *M. leprae* with the smallest genome has an estimated doubling time of 12–14 days, whereas *M. smegmatis* doubles every 3 hours. In general, the pathogenic mycobacteria are slow growers compared to the non-pathogenic species.

**Figure 1:**
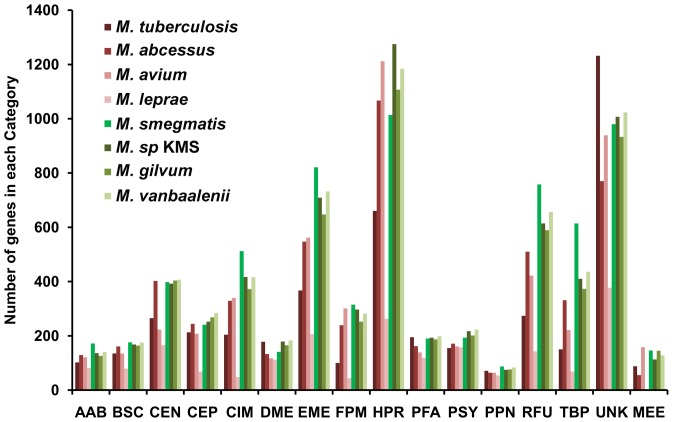
Classification of mycobacterial genes into functional classes. The genes are categorized into 16 different role categories based on the TIGR classification system, where AAB represents Amino acid and Biosynthesis; BSC- Biosynthesis of Co-factors; CEN- Cell envelope; CEP- Cellular processes; CIM- Central intermediary metabolism; DME- DNA Metabolism; EME-Energy metabolism; FPM – Fatty acid and Phospholipid metabolism; HPR – Hypothetical proteins; PFA – Protein Fate; PSY – Protein synthesis; PPN – Purine, pyrimidine and nucleotide metabolism; RFU – Regulatory functions (Regulatory functions+Transcription); TBP – Transport and Binding proteins; UNK – Unknown & Unclassified proteins; MEE – Mobile extrachromosomal elements.

The distribution of genes into various functional classes is presented in Figure 1 based on the TIGR classification system. Data is shown for 8 out of the 10 species as the TIGR classification is not available for *M. marinum* and *M. ulcerans*. Most of the genomes have a large number (∼1000) of hypothetical proteins and proteins with unknown functions (∼1000). Among the primary metabolism genes, energy metabolism has a large presence in all genomes with an average of 600 genes. Genes coding for amino acid biosynthesis are less than 200. Purine and pyrimidine metabolism related genes are also fewer in number compared to other categories (<100). Most *Mycobacterium* genomes have a large number of regulatory genes. The number of genes in some categories such as nucleotide metabolism (PPN), protein fate (PFA) and cofactor biosynthesis (BSA) are fairly constant across all species considered in this work.

A few differences can be noted between the pathogens and the non-pathogens. On average, non-pathogens have twice the number of transport and binding proteins compared to the pathogens. With more than 600 transporters, *M. smegmatis* has exceptionally high number of genes in this class. Regulatory proteins are also much larger (1.6 times) in number in non-pathogens, with highest number of regulatory proteins in *M. smegmatis*. Similarly, non-pathogens have 1.5 times the number of energy and central intermediary metabolism genes than the pathogens.

*M. leprae* with the smallest genome has reduced number of genes in most categories including regulatory and transport proteins, energy metabolism, and fatty acid metabolism. However, the number of genes in other classes like purine and pyrimidine metabolism, DNA metabolism and protein synthesis is same as other organisms.

### Variation of Gene Number with Genome Size

**Figure 2:**
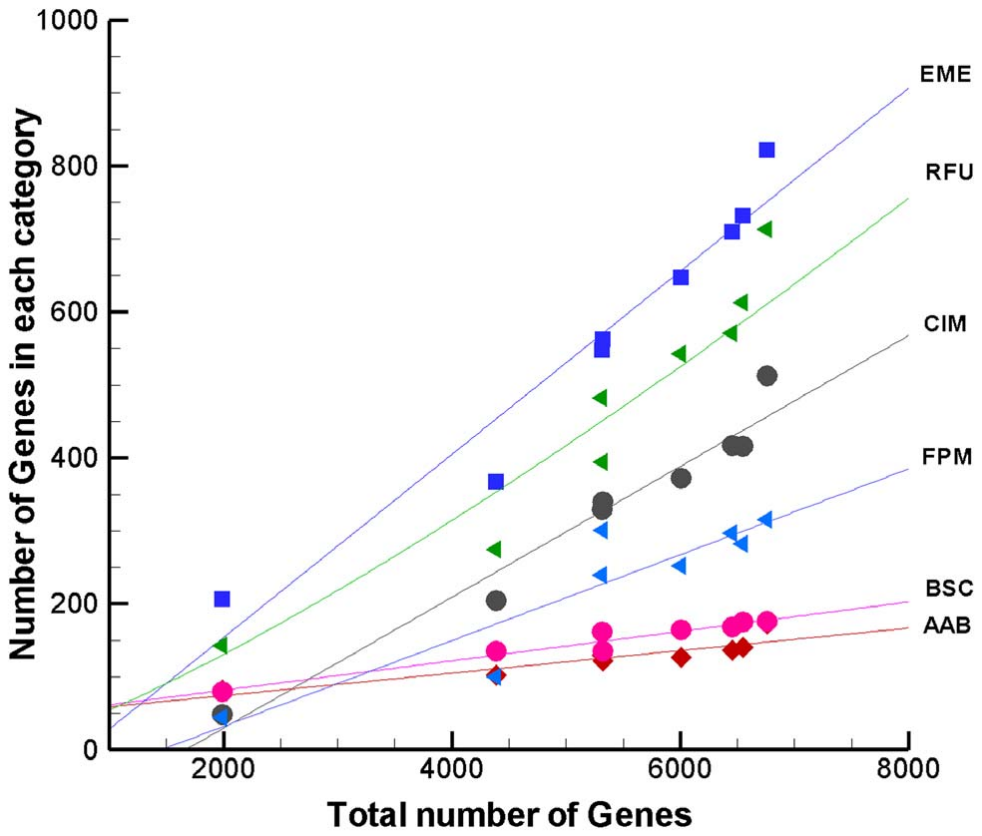
Variation of number of genes in functional categories with genome size. The role category abbreviations are same as that in Figure 1.

The number of genes in each functional group is found to increase with the total number of genes in the genome, a measure of genome size. The rate of increase is linear in most cases, and some of the representative curve-fits are shown in Figure 2. Genes coding for energy metabolism increase at a high rate with respect to the genome size. Interestingly, regulatory genes and transport proteins (data not shown) also increase at a high rate. By comparison, categories such as amino acid biosynthesis and transcription related genes increase at a slower rate. Other categories such as biosynthesis of cofactors, DNA metabolism and protein fate (not shown in the figure) also increase at a slower rate. Genes responsible for fatty acid metabolism category increase at an intermediate rate. A notable exception is *M. avium*, which has far more number of genes coding for fatty acid and phospholipid metabolism compared to that expected based on the curve fit. DNA and protein metabolism related genes do not show any significant trend with respect to genome size. The number of genes in these categories is fairly conserved across all species.

The variation of number of genes in each functional category with genome size can be explained equally well with a power fit (Figure S1). Genes related to fatty acid metabolism and central metabolism follow the power law with respect to genome size. The exponent value is 1.7 and 1.9 respectively, indicating a close to 4 times increase in the corresponding genes with every doubling of number of genes. A similar high increase is also observed for transport related genes. The exponent for mobile elements is much higher at 3.4. Genes belonging to energy metabolism, regulation and various cellular processes increase almost linearly with genome size with an exponent between 1.1–1.3.

### Identification of Orthologs

For each pair of mycobacterial genomes analyzed in this work, orthologs were identified based on a reciprocal best blast hit (RBH) approach as described in methods (see Table 2, Table S1). There is a four-fold variation in the number of orthologs, with an average of 2294 proteins common between any two species. *Mycobacterium leprae* and *Mycobacterium abscessus* have the smallest number of orthologs. The two non-pathogenic mycobacteria, *M. gilvum* and *M. vanbaalenii* share 4162 orthologs, the maximum among any two *Mycobacterium* species.

**Table 2:** Number of pair-wise orthologs between all genomes considered in this study.

*tuberculosis*										
***avium***	2413									
***ulcerans***	2466	2573								
***marinum***	2837	3008	3692							
***leprae***	1343	1269	1306	1353						
***abscessus***	1675	1909	1723	1965	1000					
***smegmatis***	2117	2605	2212	2586	1145	2177				
***KMS***	2130	2631	2225	2639	1140	2069	3536			
***vanbaalenii***	2138	2589	2254	2651	1131	2112	3562	3717		
***gilvum***	2035	2518	2149	2499	1112	2022	3275	3561	4162	
***coelicolor***	645	783	663	728	345	784	1092	969	946	908
***coli***	26	30	29	32	16	31	47	35	38	34
**Organism**	***tuberculosis***	***avium***	***ulcerans***	***marinum***	***leprae***	***abscessus***	***smegmatis***	***KMS***	***vanbaalenii***	***gilvum***
**Total # of Proteins**	3988	5120	4160	5423	1605	4920	6716	5460	5979	5241

Nomenclature: coelicolor stands for *Streptomyces ceolicolor* A3(2) and coli refers to *Escherichia coli* K12.

For other organisms, the abbreviations refer to the organism mentioned in Table 1.

Next, a similarity score is computed based on the normalized number of ortholog pairs between two genomes. This is referred to as the % shared gene content [27]. Table 3 presents the normalized scores with respect to the smaller (upper triangular matrix) or larger (lower triangular matrix) genome size of the two species being compared. The % gene content varies over a wide range with 89% shared content between *M. ulcerans* and *M. marinum* as opposed to only 17% between *M. smegmatis* and *M. leprae*. On an average, 50% of the genome is shared between any two species. The four non-pathogens considered in this study are similar to each other, with an average shared gene content of 62%. In contrast, the average gene content among pathogens is 52% and 53% of genome of pathogens has an ortholog in the non-pathogens. On the other hand, only 37% of non-pathogens' genome finds an ortholog in the pathogens. It should be noted that the larger genome size of non-pathogens compared to the pathogens, contributes to this difference.

**Table 3:** Normalized Gene Content between *Mycobacterium* species.

Organism	*tuberculosis*	*avium*	*ulcerans*	*marinum*	*leprae*	*abscessus*	*smegmatis*	*KMS*	*vanbaalenii*	*gilvum*
***tuberculosis***		61	62	71	84	42	53	53	54	51
***avium***	47		62	59	79	39	51	51	51	49
***ulcerans***	59	50		89	81	41	53	53	54	52
***marinum***	52	55	68		84	40	48	49	49	48
***leprae***	34	25	31	25		62	71	71	70	69
***abscessus***	34	37	35	36	20		44	42	43	41
***smegmatis***	32	39	33	39	17	32		65	60	62
***KMS***	39	48	41	48	21	38	53		68	68
***vanbaalenii***	36	43	38	44	19	35	53	62		79
***gilvum***	39	48	41	48	21	39	49	65	70	
**TOTAL # of Proteins**	3988	5120	4160	5423	1605	4920	6716	5460	5979	5241

* Upper triangular matrix represents % gene content defined as genes shared by two genomes/genes in smaller genome;

Lower triangular matrix represents the % gene defined as genes shared by two genomes/genes in larger genome.

Orthologs were also computed against two outgroup organisms, *E. coli* and *S. coelicolor*. As *E. coli* is phylogenetically distinct from *Mycobacterium* genus, it shares less than 50 orthologs with them. Orthologs shared with *S. coelicolor* were significantly higher as *S. coelicolor* belongs to the same actinomycetales family. However, the number of orthologs between any two *Mycobacterium* species was higher compared to that with *S. coelicolor*.

### Identification of Core Homologs

**Figure 3:**
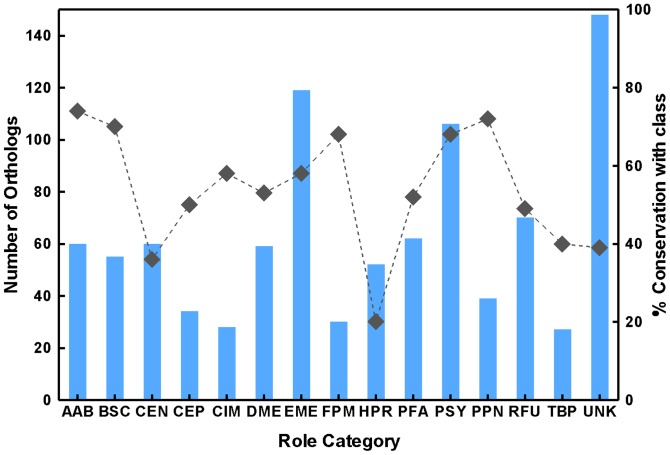
Distribution of core orthologs into various functional categories. The bar graphs represent the number of core orthologs in each category based on the TIGR annotation of the genes in *Mycobacterium leprae*. The line plot shows the % conservation of each class with respect to total number of genes in that class in *M. leprae*.

Based on the orthologs identified above a set of core genes present in all 10 species were identified. Genes included in this core list are such that, any gene has an ortholog in all other 9 *Mycobacterium* species. A set of 759 genes (Table S2) were thus identified as core orthologs. Figure 3 shows the distribution of these genes into various functional classes. *M. leprae* was chosen as the reference because it has the smallest genome and also the lowest number of genes in each category.

The core genes span all functional categories, with more than 400 genes belonging to various metabolic pathways. These include energy metabolism, amino acid biosynthesis and DNA and protein metabolism. A large number of the genes (∼200) are either conserved hypotheticals or do not have a function assigned as yet. More than 80 genes belong to various cell wall related classes, such as, transport proteins. None of the PE/PPE class genes are part of the core orthologs.

Next, the percentage of genes conserved in all 10 mycobacteria genomes was computed for each functional category, again based on *M. leprae*. Greater than 70% conservation was observed for genes related to amino acid biosynthesis, nucleotide biosynthesis and biosynthesis of cofactors. Genes related to protein synthesis and fatty acid metabolism were also fairly conserved. Genes belonging to energy metabolism and various regulatory genes show moderate conservation between 50–60%, indicating variability among different species in these pathways. The least conserved classes are those related to cell wall, such as, transport and secreted proteins and PE/PPE family proteins. It is interesting to note that the set of core orthologs does not vary much even if *M. leprae* is excluded from the comparison.

### Phylogeny Analysis

#### Phylogenetic tree based on individual genes

**Figure 4:**
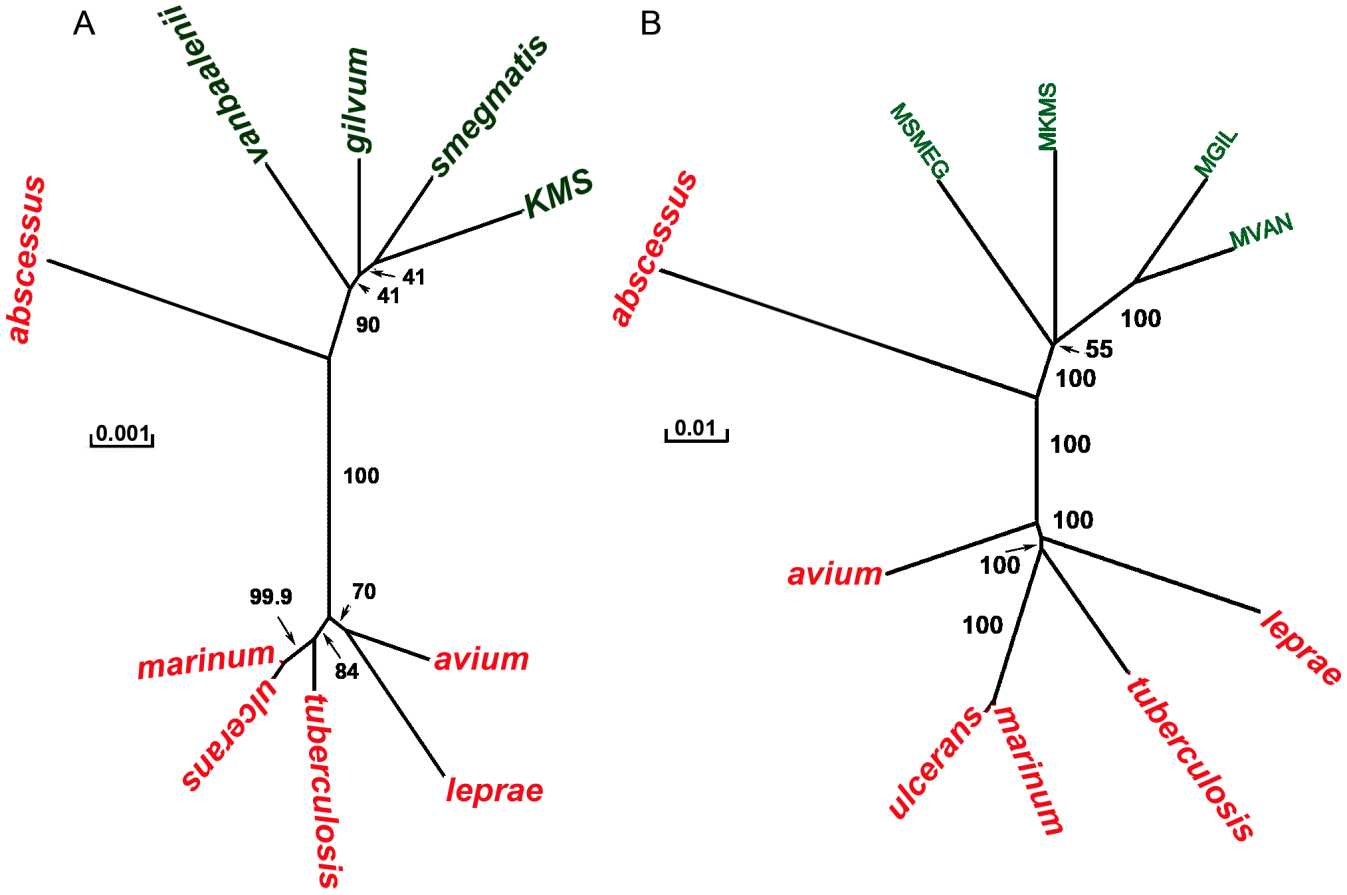
Phylogenetic tree based on nucleotide and protein gene sequence. Tree based on 16S rRNA sequence is shown in (a); whereas tree in (b) is based on concatenation of protein sequences of 759 core orthologs. The 16S tree is based on the Fitch-Margoliash method, whereas neighbour-joining method was used to draw the tree in (b).

**Figure 5:**
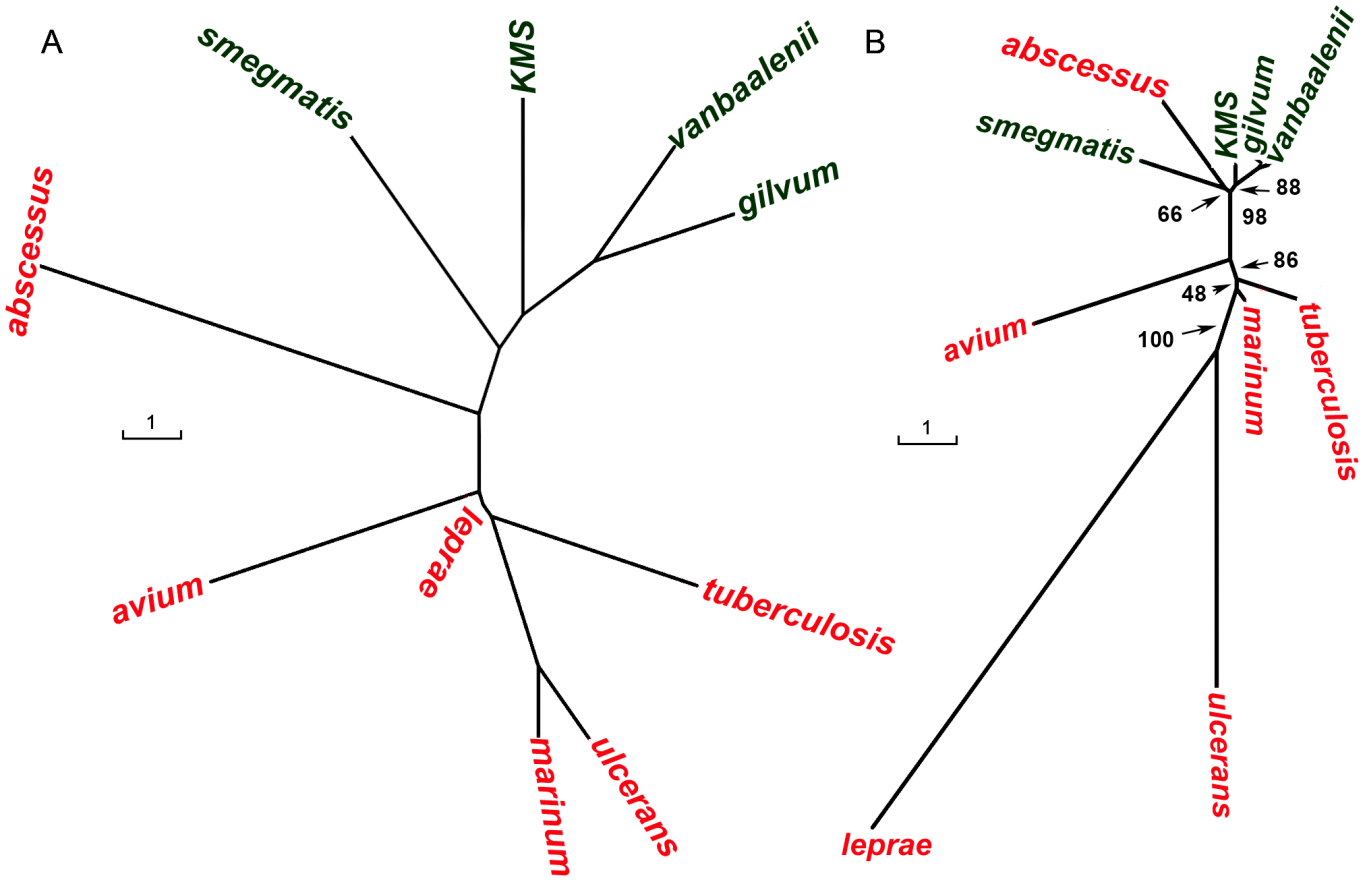
Phylogeny of mycobacteria based on gene content and synteny. Tree shown in (a) is based on number of orthologs shared among two genomes. The % similarity is based on smallest of the two genomes. Tree shown in (b) is based on order of the core orthologs in each genome. The distance between two genomes is defined as the number of reversals to convert the gene order of one genome into that of the second. The trees are based on the Fitch-Margoliash method.

Next, the phylogenetic relationship between the 10 *Mycobacterium* species is explored and the results are presented in Figures 4 and 5. Additional trees based on the various tree building methods are shown in Figure S2. The trees with inclusion of an outgroup are also presented.

A phylogenetic tree was constructed based on the 16S nucleotide sequence. Figure 4a shows the consensus tree based on the Fitch-Margoliash method. All the pathogenic mycobacteria cluster together with *M. marinum*, *M. ulcerans* and *M. tuberculosis* forming a close group. Similarly, the non-pathogenic *Mycobacterium* species also lie close to each other. *M. abscessus* lies at the boundary of the two groups. It is closer to non-pathogens than to the pathogens.

A phylogenetic tree was also constructed based on the nucleotide sequence of dnaN, a DNA polymerase gene that is conserved across all the genomes. The tree is shown in Figure S3a. Similar to the 16S based tree, the pathogenic and non-pathogenic mycobacteria form two distinct groups, but the distances between various species is higher as compared to those in Figure 4a. This reflects a higher conservation of 16S sequences than that of dnaN. Also, the bootstrap values at many branch points indicate higher statistical significance based on the dnaN tree.

#### Phylogenetic tree based on Core Genes' Sequence

Next, a phylogenetic tree was constructed based on the protein sequence of all the 759 core orthologs as described in the methods section. The phylogenetic tree, shown in Figure 4b, is remarkably similar to those discussed above. Compared to the 16S based tree, this tree is better resolved. Two sub-groups are identified in the tree; one comprising of the pathogenic species and the other comprising of the non-pathogenic species. *M. abscessus* forms a separate clade, and is closer to non-pathogens than to pathogens. Also, *M. avium* is further from the other pathogenic mycobacteria. Similar observation is also made in the dnaN based tree.

#### Phylogenetic tree based on gene content

The phylogenetic tree shown in Figure 5a is based on gene content rather than sequence similarity. The smaller genome which defines the maximum possible shared orthologs is used as a reference genome to compute % gene content. Remarkably, the pathogens and non-pathogens once again form two separate groups. Among the non-pathogens, *M. vanbaaleni* and *M. gilvum* are closest, whereas *M. marinum* and *M. ulcerans* are closest among the pathogens. As observed in previous trees, *M. abscessus* is at the border. However, it is equidistant from the pathogens and the non-pathogens.

Alternate definitions of distance between two genomes were also implemented. For example, for the phylogenetic tree shown in Figure S3b, distance between any two genomes is computed based on the percentage of larger genome conserved between two organisms. The tree is similar to the earlier case except that *M. leprae* is further from all other genomes, a consequence of its small genome size.

#### Phylogenetic Tree based on Gene Order

The order of genes on the genome also provides an indication of the evolutionary distance between organisms. Therefore, a phylogenetic tree was constructed based on the conservation of the order of the core orthologous genes on the genome. The gene order tree in Figure 5b shows a few distinct differences from the other trees. *M. abscessus* groups with the non-pathogens instead of being at the border. Second, *M. leprae* is most distant from any other *Mycobacterium* species indicating that there have been many rearrangements in its genome even for the core orthologous genes.

It is also interesting to note that the gene order is more conserved among the non-pathogens as compared to the pathogens. For example, the three species, *M. KMS*, *M. vanbaalenii* and *M. gilvum* are closely spaced on the tree. Note that based on the 16S tree, it cannot be inferred that the non-pathogens are very similar. Among the pathogens, *M. tuberculosis* and *M. marinum* have similar gene order whereas others are distant. Also, although the 16S sequence of *M. marinum* and *M. ulcerans* is highly conserved, the order of genes is not. Overall, the pathogens and non-pathogens form two separate clades.

Note that the order of genes on *M. smegmatis* genome was considered as the reference. However, changing the reference does not affect the distance between any two genomes.

### Gene Synteny Analysis

**Figure 6:**
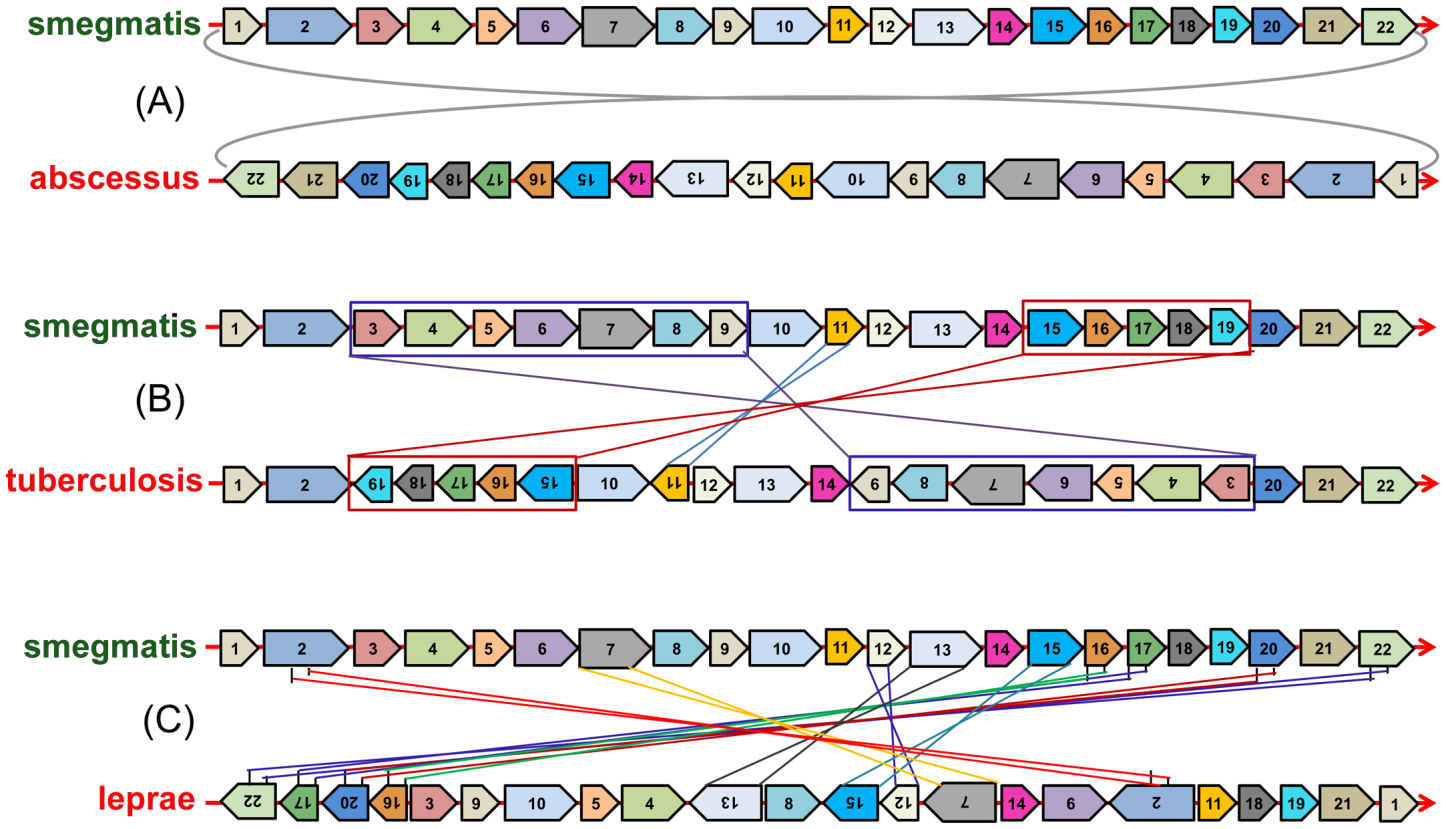
Synteny Analysis of Core Orthologs in mycobacteria. Order of blocks in few chosen mycobacteria are shown. Three representative rearrangement patterns are shown (A–C) for 22 blocks where each block had a minimum of 10 genes.

Next, a detailed analysis of gene order was performed to analyse the rearrangement pattern in the *Mycobacterium* genomes. The core genes are grouped into synteny blocks as described in methods. A total of 95 blocks were found with the number of genes in each block varying from 1 to 40. 22 blocks with a minimum of 10 genes are considered for visualization. Figure 6 shows the order of these blocks with respect to *M. smegmatis* in three representative genomes to illustrate the increasing complexity of rearrangements.

The relative order of the blocks is conserved in most organisms. In particular, *M. KMS* and *M. vanbaaleni* genomes are completely conserved in both relative order and gene orientation (see Figure S4). Other genomes show conservation of relative position of the blocks, but a reversal of gene orientation. For example, genes in block 11 are flipped in direction in *M. marinum* with respect to *M. smegmatis*. Also, the *M. abscessus* genome shows complete reversal of gene orientation compared to the *M. smegmatis* reference, but the relative order of the 22 different blocks is maintained. In the *M. gilvum* genome, blocks 1–10 and blocks 12–22 form two sections that are flipped in orientation with the relative order of the blocks maintained. A similar scenario is observed in *M. avium*.

The rearrangement pattern in the genomes of *M. tuberculosis*, *M. ulcerans* and *M. leprae* is markedly different from that of the organisms discussed above. In all these genomes, the relative order of the blocks is different from that in *M. smegmatis*. For example, in the *M. tuberculosis* genome, three sets of blocks can be identified that have both reversed in orientation and also rearranged their position on the genome. *M. leprae* and *M. ulcerans* also show major rearrangements in their synteny blocks as compared to *M. smegmatis*. The major genes in each block are listed in the Table S3.

The phylogenetic tree in Figure 5b suggests significant rearrangements in *M. avium* with respect to both the non-pathogens and pathogens. However, on combining the genes into synteny blocks, the data shows reversal of only two segments on the genome with respect to *M. smegmatis*. The first segment consists of blocks 1–10, and the second segment consists of blocks 12–22. Block 11 in between is maintained in position and gene order.

**Figure 7:**
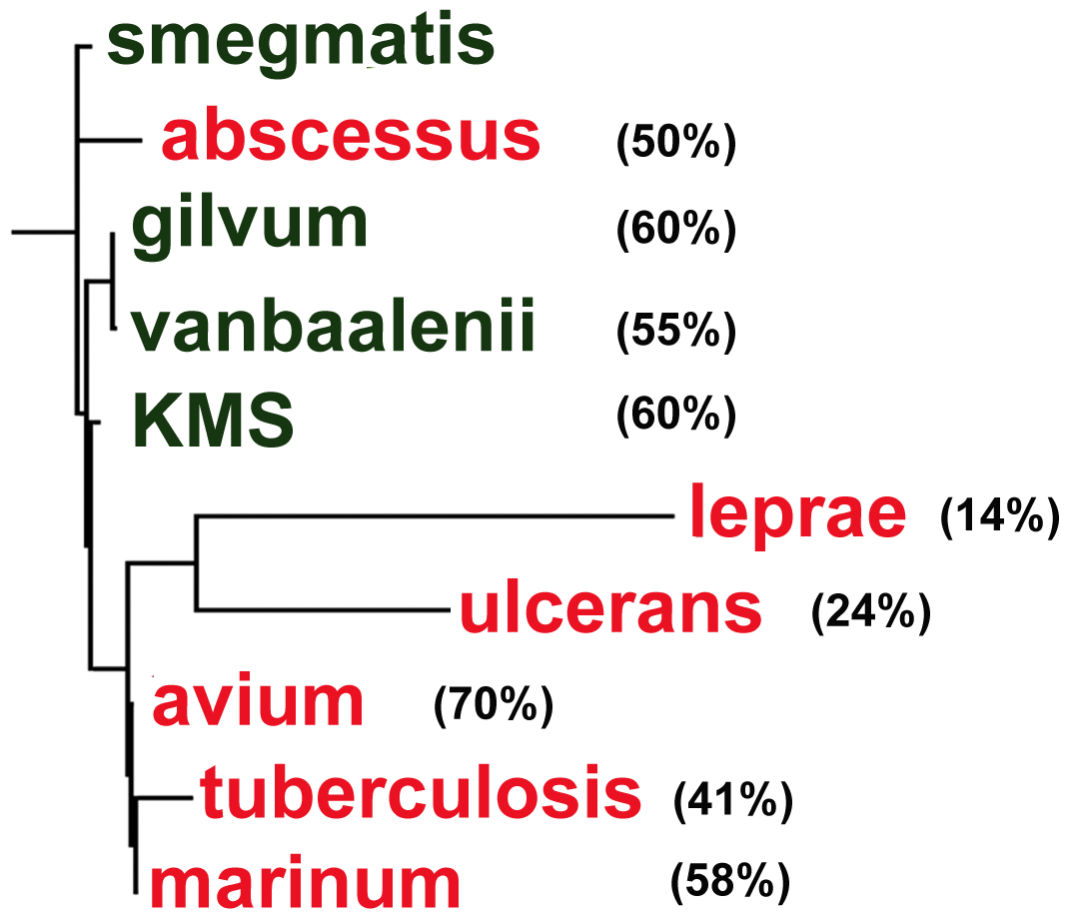
Phylogenetic tree based on order of syntenic blocks from core ortholog genes. The number in bracket denotes the % micro-rearrangements with respect to *M. smegmatis*. Pathogens are shown in red whereas green denotes non-pathogens.

To understand this further, we compared the rearrangement pattern of all 759 core orthologs with that of the synteny blocks. Percentage micro-rearrangements between any two genomes was computed based on the difference in the number of reversals for all the core orthologs considered separately and the number of reversals computed when they are considered as part of synteny blocks. Figure 7 shows the phylogenetic tree based on the synteny blocks. The computed percentages with respect to the *M. smegmatis* genome are also shown. Comparison of the core genes between *M. avium* and *M. smegmatis* show 24 reversals in gene order. However, the number of reversals decreases to 7 when the core genes on the two genomes are grouped into blocks; a reduction of 70% compared to the gene level rearrangements. This implies that there are more micro-rearrangements within blocks in the *M. avium* genome than global rearrangements. On an average, 50–60% of reversals can be classified as micro-rearrangements for most genomes. Notable exceptions are *M. ulcerans* and *M. leprae*, where only 14% and 24% rearrangements respectively are within blocks. The changes are mostly global as illustrated in Figure 6, where *M. leprae* blocks are shuffled across the genome. A similar scenario can be observed in the case of *M. ulcerans* (Figure S4).

It is interesting to note that based on the phylogenetic tree of Figure 5b *M. avium* is more distant from *M. smegmatis* as compared to *M. tuberculosis*. However, the former has mostly (70%) micro rearrangements whereas *M. tuberculosis* has more global changes, with only 41% micro-rearrangements. Correspondingly, with respect to *M. smegmatis*, the phylogenetic position of *M. avium* and *M. tuberculosis* has reversed on the gene order tree based on the synteny blocks (Figure 7).

### Genes specific to pathogens/non-pathogens

The set of orthologs identified in this study were next investigated to identify genes that are present exclusively in pathogens and non-pathogens. 9 genes were found to be present only in pathogens with no corresponding ortholog in the non-pathogens that satisfied the cut-off criteria as defined in methods section. All 9 genes had a corresponding homolog with similar function in the non-pathogens, but the sequence homology was less than 50% at protein level. In addition, the nucleotide sequences have very little homology. See Figure S5 for identity and % coverage of the *M. tuberculosis* gene with the corresponding homolog in the non-pathogens. Multiple sequence alignment of all the 9 genes in each of the 10 genomes is shown in Figure S6. It is clear that the protein sequence in the pathogens and non-pathogens is distinct.

To investigate the significance of each of the 9 genes specific to pathogens, we queried them on the TBDB database which has a compilation of many publicly available microarray datasets for *M. tuberculosis*. Interestingly, all 9 genes are found to be differentially expressed at mRNA level in *M. tuberculosis* H37Rv in response to various chemicals and antibiotics. For example, pyrE (Rv0382c) is an orotate phosphoribosyltransferase enzyme, involved in the reversible conversion of orotate to orotidine-5 phosphate in pyrimidine biosynthesis. The gene is up-regulated under antibiotic stress and down-regulated in response to starvation [30]. Up-regulation is also observed in many important mutants, such as, *dosR* and sigma factor, *sigE*. The data for all 9 genes is summarized in Table 4. Further, the protein and nucleotide sequences of these genes were queried to shed light on their plausible origin. Interestingly, while 7 genes had hits in the Corynebacterinae suborder, two genes had no significant hits other than mycobacterial pathogens. The results are summarized in Figure S7.

**Table 4:** Genes identified as exclusive to pathogens.

LOCUS	PRODUCT	*Transcriptome data for *M. tuberculosis* H37Rv	SIGNIFICANCE OF GENEFAMILY
**Rv0234c, ML2573, MMAR_0490, MAV_4936, MUL_1153, MAB_3471.**	succinate-semialdehyde dehydrogenase (*gabD1*)	NGA (4.862), STREP (−3.023), DETA (−2.229)	MTB lacks KDH activity in TCA cycle; hence, α-ketoglutarate cannot be converted to succinate. As an alternate pathway, kgd and *gabD1* help in achieving this conversion *via* utilization of glutamate [42].
**Rv0364, ML0287, MMAR_0668, MAV_4785, MUL_0104, MAB_4661.**	Conserved membrane protein	KCN (−4.291), CPM (−3.936), Sediment *vs* pellicle (−3.899)	These genes belong to DedA protein family, that are involved in membrane homeostasis [43].
**Rv0382c, ML2487, MMAR_0649, MAV_4790, MUL_0123, MAB_4260c.**	Orotatephosphoribosyltransferase (*pyrE*)	STREP (6.698), PMA (5.205), DETA (−4.493), Starvation (−5.575)	These genes are involved in the reversible conversion of orotate to orotidine-5 phosphate in the utilization of L-glutamine, essentially in pyrimidine metabolism.
**Rv0451c, ML2377, MMAR_0772,**	Membrane	Iron (2.959), OA (−2.55),	In general, MmpS proteins act as a scaffold for coupled
**MAV_3247, MUL_1935, MAB_4117c.**	protein (*mmpS4*)	WT *vs* dosT mutant (−4.224)	biosynthesis of lipid and transport machinery [44].
**Rv1404, ML0550, MMAR_2213,**	Transcriptional	DM+PMA (6.348), KCN+DETA (4.27), Rv *vs* PDIM mutant (8.806),	Rv1404 belongs to marR family of proteins. This gene acts as a repressor of two methyltransferases Rv1403c
**MAV_3374, MUL_1800, MAB_1021.**	regulator	Grown in macrophage(−4.014)	and Rv1405c. Rv1404 was found to be an important regulator of genes in response of MTB to acid shock [34].
**Rv1524, ML2348, MMAR_2353, MAV_3258, MUL_1529, MAB_4112c.**	Glycosyl transferase	Reaeration of Rv under hypoxia (2.753), 3 mM ACE (−3.577), MEN (−6.26)	Glycosyltransferases are the enzymes that synthesizeoligosaccharides,and lipopoly-sachharides [45].
**Rv3484, ML2247, MMAR_4966, MAV_0673, MUL_4040, MAB_0560.**	Hypothetical protein cpsA	OA (6.453), LA (7.257), PMA (2.882), Rv *vs* CDC1551 (−4.323), Rv *vs sigE* mutant (−3.959)	Role of cpsA protein is unknown in MTB. However, in streptococcus, this gene is involved in regulatory control ofcell wall related processes and response to antimicrobial stress [46].
**Rv3631, ML0207, MMAR_5131,**	Glycosyl	Rv *vs* DOS (2.493), Rv *vs* 9802501 (2.652), Grown in macrophage (−4.173),	Rv3631 encodes glycosyltransferases (ppgS). Catalytic activity of ppgS was found to be increased by 40–50 fold
**MAV_0525, MUL_4208, MAB_0505c.**	transferase	PMA+OA (−2.605), PMA (−3.774)	by co-transcription of Rv3632.
**Rv3632, ML0208, MMAR_5132,**	Conserved	SNG+CPM (9.742), Rv*vs*DOS (−6.406),	Rv3631 and Rv3632 are jointly involved in modification
**MAV_0523, MUL_4209, MAB_0504c.**	membrane protein	DCH(−7.865), AA (−6.174)	of cell wall component called arabidogalactan [47].

* The values shown are log_2_ ratios of condition with respect to their control. Data is from TB database located at www.tbdb.org [30].

* Expansions: NGA - Nordihydroguaiaretic acid; STREP –Streptomycin; PMA-Palmitate; DM-Defined Media; KCN-Potassium cyanide; DETA- Diethylenetriamine; LA - Linoleic acid; OA - oleic acid; Rv – H37Rv strain; SNG- S-nitrosoglutathione; CPM-Chlorpromazine; ACE – Acetate; MEN-Menadione; DCH – Dicyclohexylcarboxamide; AA - Arachidonic acid; DOS – dosS/dosT mutant; CSP – Cell surface physiology; PS – Polysaccharide synthesis; CWP – Cell wall processing; KDH-alpha-ketoglutarate dehydrogenase; kgd -alpha-ketoglutarate decarboxylase.

The non-pathogens have close to 400 genes that have been lost from the pathogens (see Table S4 for detailed list). None of these genes have a homolog in any of the pathogens at both the nucleotide and protein level. A large fraction of these genes are either hypothetical proteins or have not been assigned a function yet. 17% genes are classified as transport and binding proteins, most of which are ABC transporters. The other major functional class represented in these 400 genes is regulation. Majority of the regulatory genes are transcriptional regulators encompassing AraC, ArsR, MarR, CadC, LuxR families and a few anti-sigma factors. All four non-pathogenic strains considered in this study are soil bacteria. Many of these genes are therefore specific for survival in soil environment.

## Discussion

In this work, the genomes of 10 representative *Mycobacterium* species are compared. The chosen set consist of both pathogenic and non-pathogenic species. Most of the comparative genomic studies so far are limited to either strains/species belonging to the *Mycobacterium tuberculosis* complex or to a comparison between two *Mycobacterium* genomes. In this study, we have focussed on the overall difference between pathogens and non-pathogens to shed light on some common aspects of pathogenesis.

The genome size of *Mycobacterium* species considered for analysis varies from 2 Mb to 7 Mb. We have studied the distribution of genes into various functional categories with varying genome size. Most gene families follow the linear or power law variation with respect to the total number of genes. The general trends are similar to that reported earlier by Koonin and Wolf [31], where a few COG functional classes were studied and the number of proteins was found to be correlated with the genome size. A power law function was used to explain this relationship and the exponent varied from 0.1 for translation related genes to 1.6 for transcription regulators. In our analysis, the regulatory genes scale as 1.3^th^ power of the total number of proteins in the genome. Genes coding for protein synthesis are found to be fairly constant across all mycobacteria. By comparison, Konstantinos and James [32], reported a reduction in the % of genome coding for translation with increasing genome size. The linear variation of the number of genes with respect to genome size is independent of the classification system (see Figure S8).

Orthologs between all mycobacterial species were determined pairwise using a reciprocal best blast hit approach. It was found that on average 50% genes in any genome are conserved in other species. We identified a set of 759 proteins shared among all mycobacteria. These include primary metabolism related genes and a large number of hypothetical proteins. A large number of genes present in all non-pathogens have been lost from the pathogens. In contrast, only 9 genes are exclusively present in all pathogens. Although these genes had a functional homolog in the non-pathogens, the sequence was considerably diverse. Many of these genes have been implicated in virulence. For example, Rv1404 is a transcriptional regulator for the methyltransferase Rv1405c which is found to be essential for survival in a mouse infection model [33], [34]. Further experiments are necessary to elucidate the role of these 9 genes in pathogenesis.

The phylogenetic relationship between the *Mycobacterium* species presented above is based on four different methods. The first approach uses the sequence of the house-keeping genes, 16S and dnaN. It is interesting to note that compared to 16S, the tree based on dnaN nucleotide sequence is better resolved and has higher bootstrap support at many branches. The other three methods, namely, gene content, core genes sequence and gene order utilize whole genome information. The trees based on the sequence-based methods clearly separate the pathogenic and non-pathogenic mycobacteria into two different clades. Among the pathogens, *M. marinum* and *M. ulcerans* have very different disease causing potential; however most sequence based methods show them to be very similar [35]. The gene content based tree and the gene order tree resolve the differences between these two organisms.

Gene order based phylogeny is distinct from the sequence based methods as gene order is expected to change in a non-clock like manner. It may therefore be more suited to resolve both very short and very long branches [36], [37], [38]. For example, gene order based phylogeny in proclorococcuus [39] and methanogens [40] resulted in a different topology as compared to the one based on sequence. In our work, a disease-causing species *M. abscessus* is grouped with the non-pathogens in the gene order based tree. In all other trees based on gene sequence, *M. abscessus* forms a separate clade between pathogens and non-pathogens. This is probably because the gene order phylogeny groups the organisms into rapid and slow-growers. *M. abscessus* is closer to the non-pathogens in terms of its growth characteristics [41], and therefore is placed away from the pathogens.

In summary, phylogenomics of mycobacteria presented in this work illustrates the evolution of pathogenic mycobacteria through genome reduction and sequence diversity. In addition to being distinct in terms of gene sequence, pathogenic genomes have also undergone significant rearrangements in gene order compared to the non-pathogens. Comparative analysis using a combination of genomic features provided better separation among the *Mycobacterium* genomes. This approach has the potential to resolve the evolution of closely related species such as *M. tuberculosis* complex genomes, and is currently being extended to include additional sequenced *Mycobacterium* genomes.

## Supporting Information

Figure S1 **Variation of number of genes in different functional categories with genome size.** The variation is described as a power law function.
(TIF)

Figure S2 **Compilation of phylogenetic trees based on different tree-making algorithms.** The corresponding bootstrap values are also shown.
(PDF)

Figure S3 **Phylogenetic relationship of pathogens and non-pathogens based on (a) dnaN nucleotide sequence, (b) Gene content normalized by larger genome.** The branch lengths in the dnaN tree are based on the Fitch-Margoliash method.
(TIF)

Figure S4 **Rearrangement pattern of synteny blocks based on core orthologs in mycobacteria genomes.** Organisms not shown in Figure 6 are represented here.
(TIF)

Figure S5 **BLAST hits for exclusive pathogenic genes in each of the non-pathogens considered in this study.** The exclusive genes in *M. tuberculosis* H37Rv were used as reference for BLAST.
(PDF)

Figure S6 **Multiple sequence alignment of all 9 exclusive genes of pathogens.** File shows the multiple sequence alignment for each of the 9 exclusive genes present in pathogen for all the ten organisms considered in this study. Conservation of amino acid residue is represented by (.), similarly the gaps are shown by (−). Specially shaded regions indicate the conserved domain of protein families.
(PDF)

Figure S7 **BLAST results to determine plausible origin of genes exclusive to pathogens.**
(PDF)

Figure S8 **Comparison of results based on genomic features with McGuire** ***et. al*** **.**
(PDF)

Table S1 **Orthologs of all** ***Mycobacterium*** **through pairwise comparison.** Supplementary excel file contains all pairwise orthologs. Each worksheet corresponds to a pair of genomes. For example, Sheet named ‘MSMEG_MTB’ lists the number of orthologs found between *M.smegmatis* and *M.tuberculosis* H37Rv. Each pair of numbers represents the orthologous gene pair. For example 1–2 represents MSMEG_0001 is an ortholog for Rv0002.
(XLSX)

Table S2 **List of core orthologs with attributes.** Supplementary excel file lists the gene id's of 759 core orthologs in all mycobacteria. Gene name, annotation, role and subrole for each entry of *M.leprae* are shown. Corresponding orthologs from other 9 organisms are also listed. Pathogens are shaded in red and green shade represents non-pathogens.
(XLSX)

Table S3 **List and details of genes present in 22 blocks.** Supplementary excel file shows list of genes encompassing 22 blocks in all 10 organisms. First column shows the block number and each block is shaded with different color. Gene name, annotation, role and subrole are shown for each entry based on *M. smegmatis* as the reference. Start coordinates, Strand and Locus for each organism are also shown.
(XLSX)

Table S4 **Genes common to all non-pathogens but absent in pathogens.** Supplementary excel file shows the genes common to all non-pathogens. 392 genes were found to be common among non-pathogens. *M. smegmatis* was used as the representative organism to show gene name, annotation, upto 3 possible mainrole and subroles. The corresponding ortholog in other non-pathogens are also listed. In addition to that, a pivot table is given to show the distribution of genes into different role categories.
(XLSX)
