# Supplementary figures and images for: Comparative Phylogenomics of Pathogenic and Non-Pathogenic Mycobacterium
Source: PLoS One. 2013 Aug 28;8(8):e71248. doi: 10.1371/journal.pone.0071248 (PMC3756022; doi:10.1371/journal.pone.0071248)

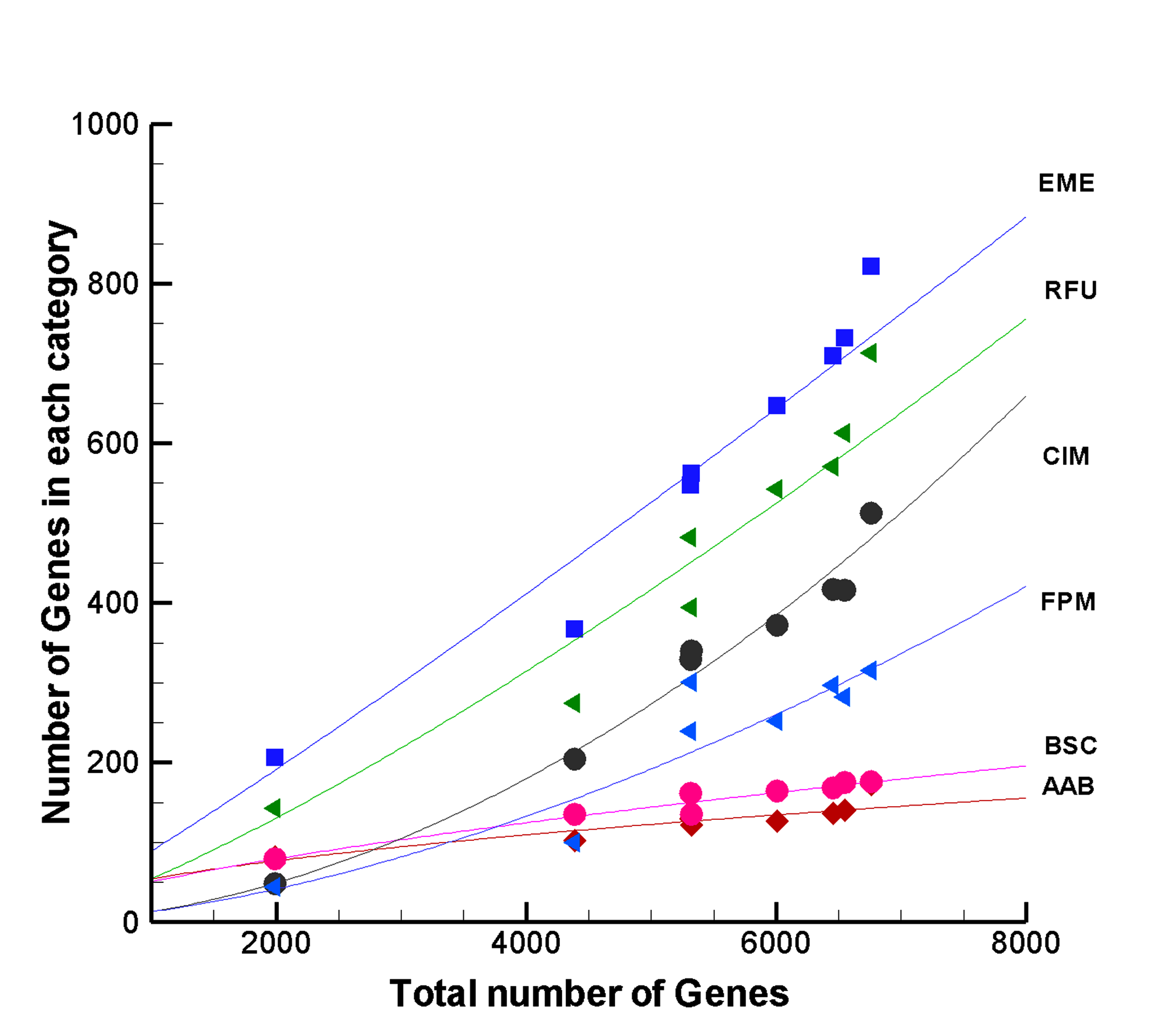

Supplement: Figure S1 — Variation of number of genes in different functional categories with genome size. The variation is described as a power law function. (TIF) [file pone.0071248.s001.tif]

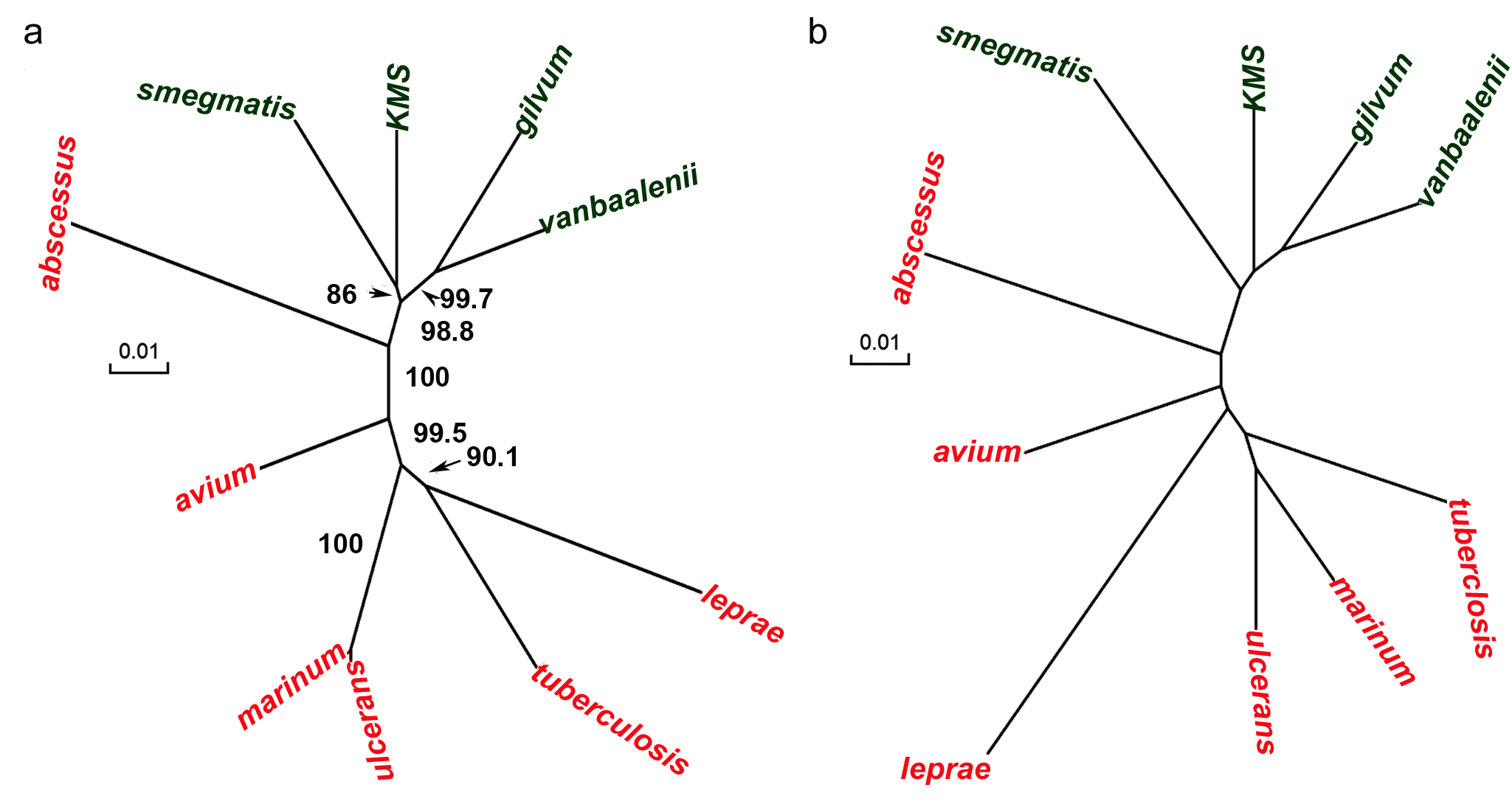

Supplement: Figure S3 — Phylogenetic relationship of pathogens and non-pathogens based on (a) dnaN nucleotide sequence, (b) Gene content normalized by larger genome. The branch lengths in the dnaN tree are based on the Fitch-Margoliash method. (TIF) [file pone.0071248.s003.tif]

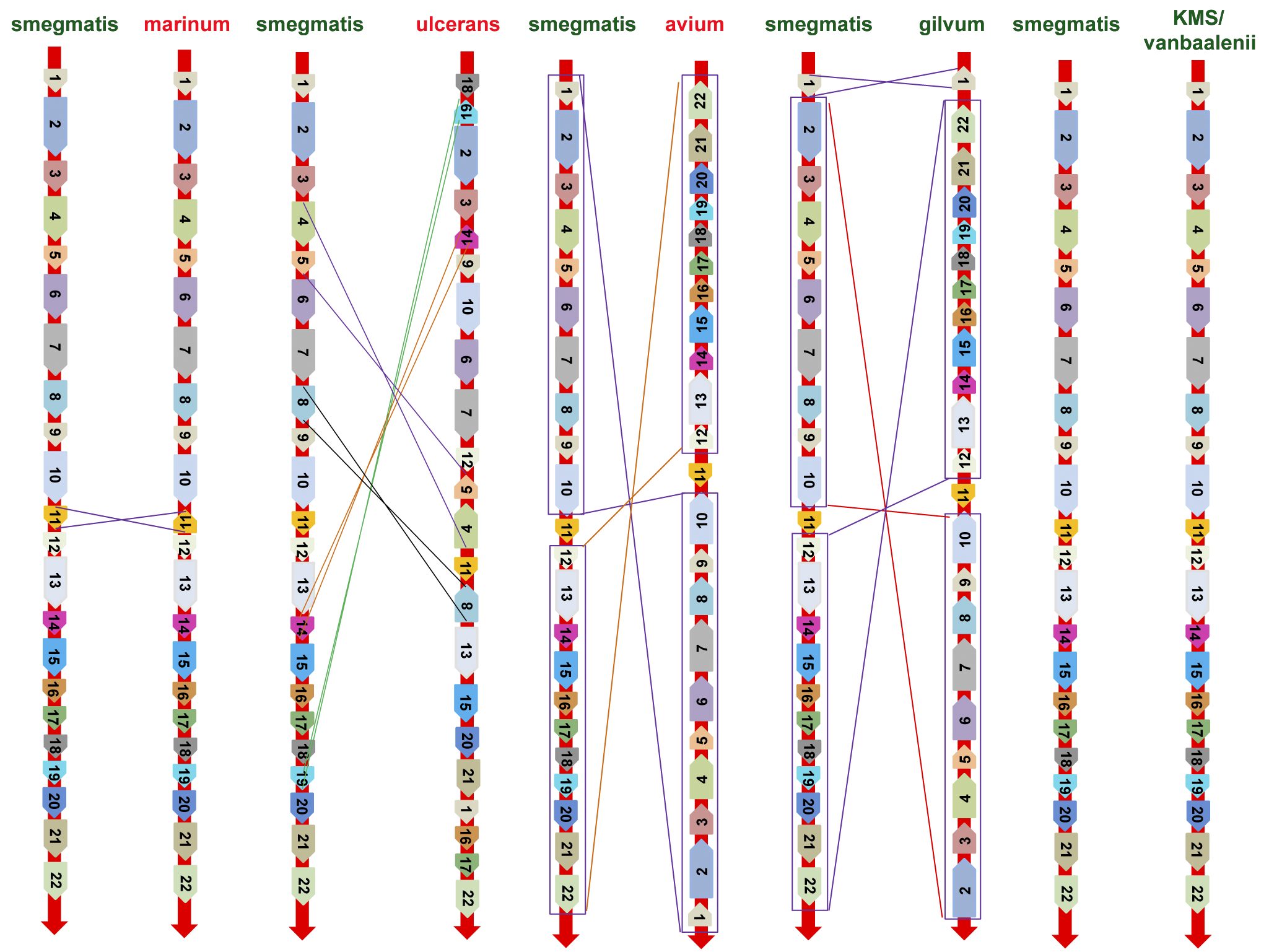

Supplement: Figure S4 — Rearrangement pattern of synteny blocks based on core orthologs in mycobacteria genomes. Organisms not shown in Figure 6 are represented here. (TIF) [file pone.0071248.s004.tif]
