# Supplementary material for: Comparative Phylogenomics of Pathogenic and Non-Pathogenic Mycobacterium
Source: PLoS One. 2013 Aug 28;8(8):e71248. doi: 10.1371/journal.pone.0071248 (PMC3756022; doi:10.1371/journal.pone.0071248)
Supplement: Figure S2 — Compilation of phylogenetic trees based on different tree-making algorithms. The corresponding bootstrap values are also shown. (PDF) [file pone.0071248.s002.pdf]

Phylogenetic tree of 16S rRNA

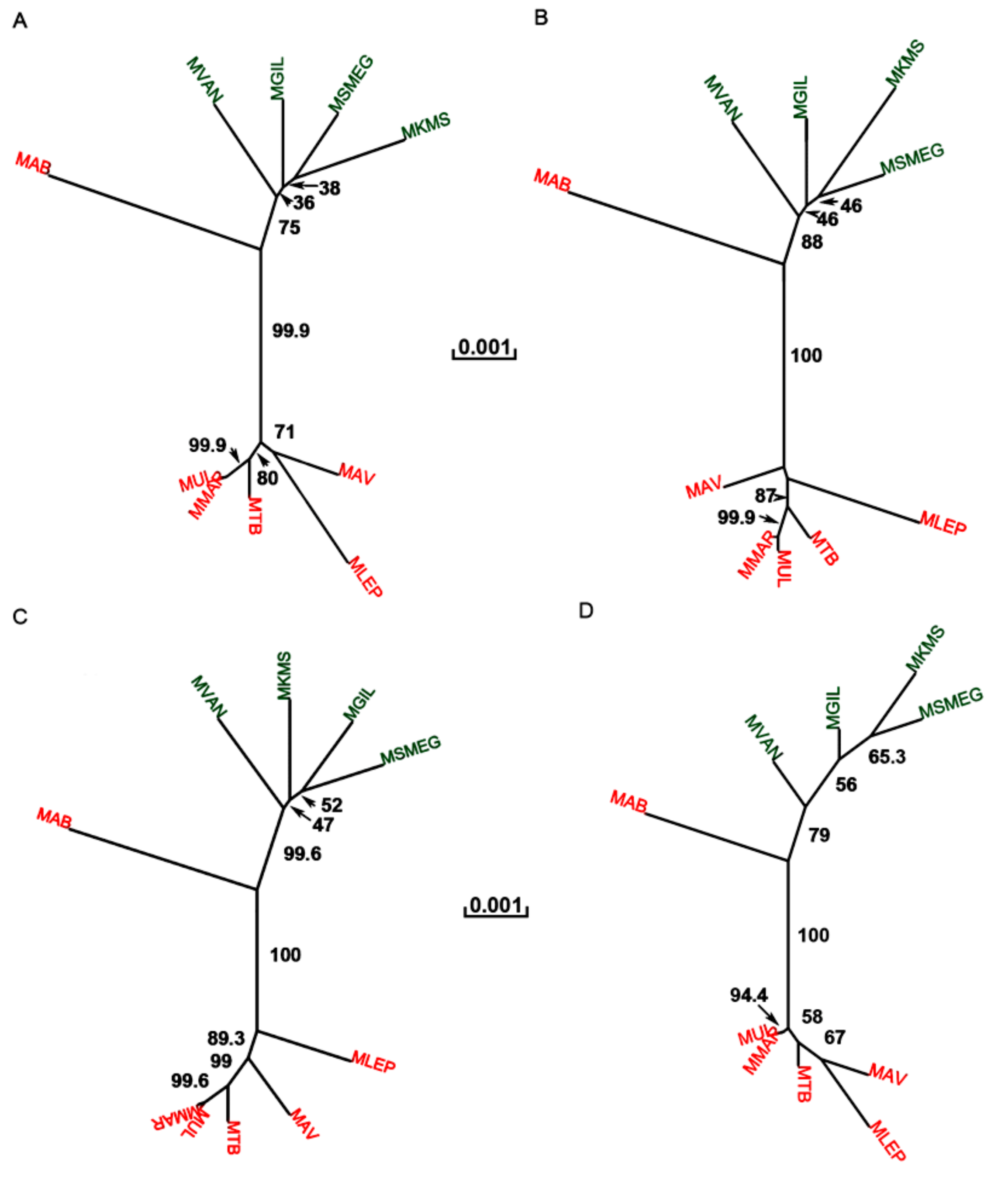

Figure shows the phylogenetic tree for 16s rRNA drawn by following methods: A) Fitch-Minimum evolution, B) Neighbor-Joining, C) UPGMA, D) Maximum likelihood. The numbers in branches indicate the bootstrap percentage values for 1000 replicates.

**Nomenclatures: MTB- *M. tuberculsis* H37Rv; MAB - *M. abscessus* ATCC 19977; MAV - *M. avium* 104; MLEP - *M. leprae* TN; MUL - *M. ulcerans* Agy99; MMAR - *M. marinum* ; MSMEG- *M. smegmatis* mc^2 155; MKMS – *Mycobacterium sp*. KMS; MGIL- *M. gilvum* PYR-GCK; MVAN - *M. vanbaaleni* PYR-1. Names colored in red indicate pathogens and green color represents non-pathogens.

Phylogenetic tree based on concatenation of Core gene sequences

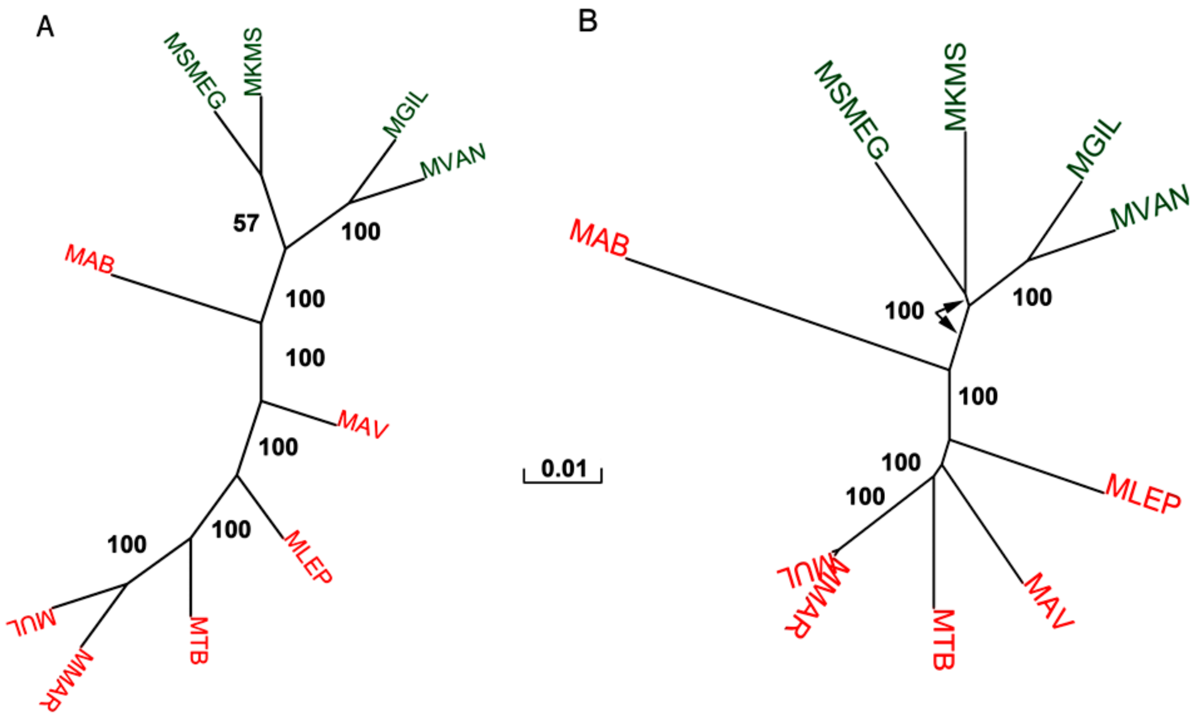

Figure shows the phylogenetic tree based on concatenation of 759 core gene sequences drawn by following methods: A) Minimum evolution, B) UPGMA. Trees were generated using MEGA v5.1. The numbers in branches indicate the bootstrap percentage values for 1000 replicates.

Phylogenetic tree based on Gene Order

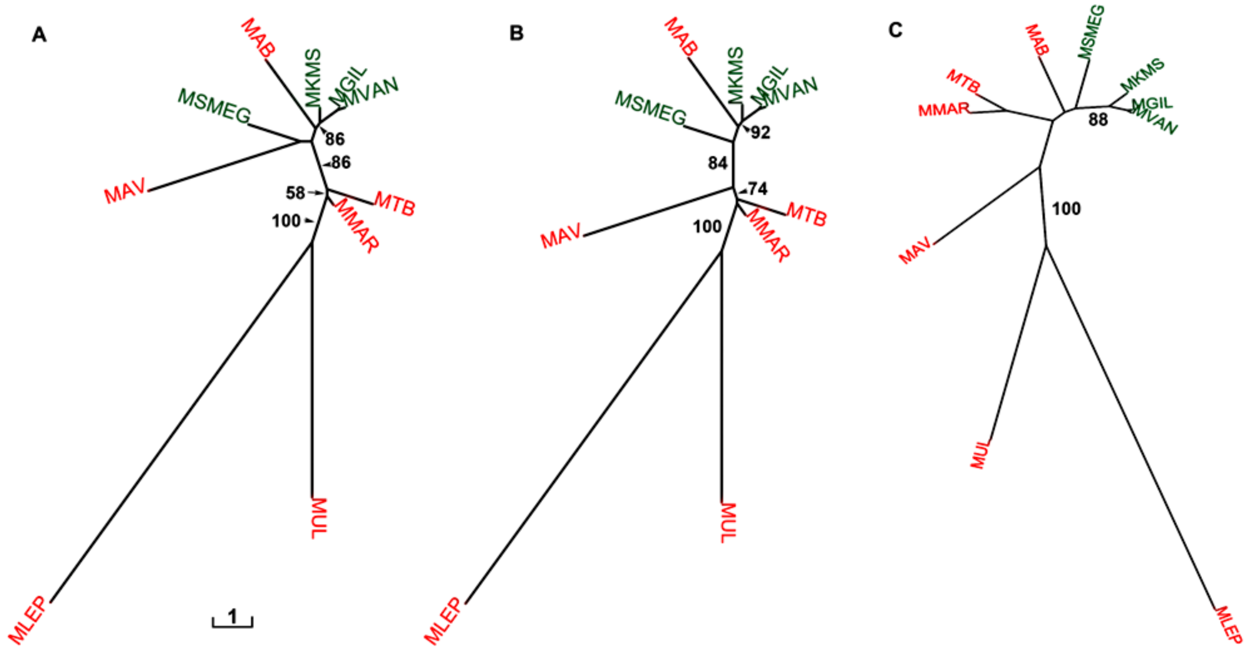

Figure shows the phylogenetic tree for gene order based on 759 core orthologs sequences. Trees shown here are drawn by following methods: A) Fitch-Minimum evolution, B) Neighbor-Joining, C) UPGMA. Numbers near branches indicate the jacknife values for 50 sets.

Phylogenetic tree based on Gene content (Smallest genome)

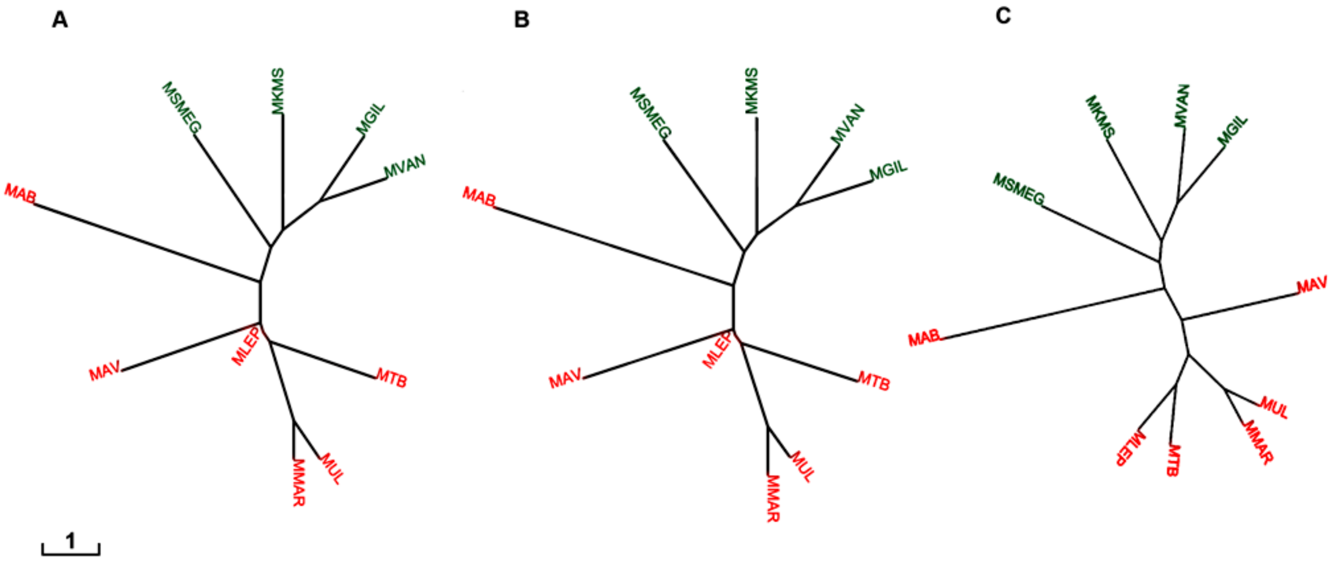

Figure shows the phylogenetic tree for gene content based on % similarity of orthologs computed by smallest of two genomes. Trees shown here are drawn by following methods: A) Fitch-Minimum evolution, B) Neighbor-Joining, C) UPGMA

Phylogenetic tree of *dnaN* nucleotide sequences

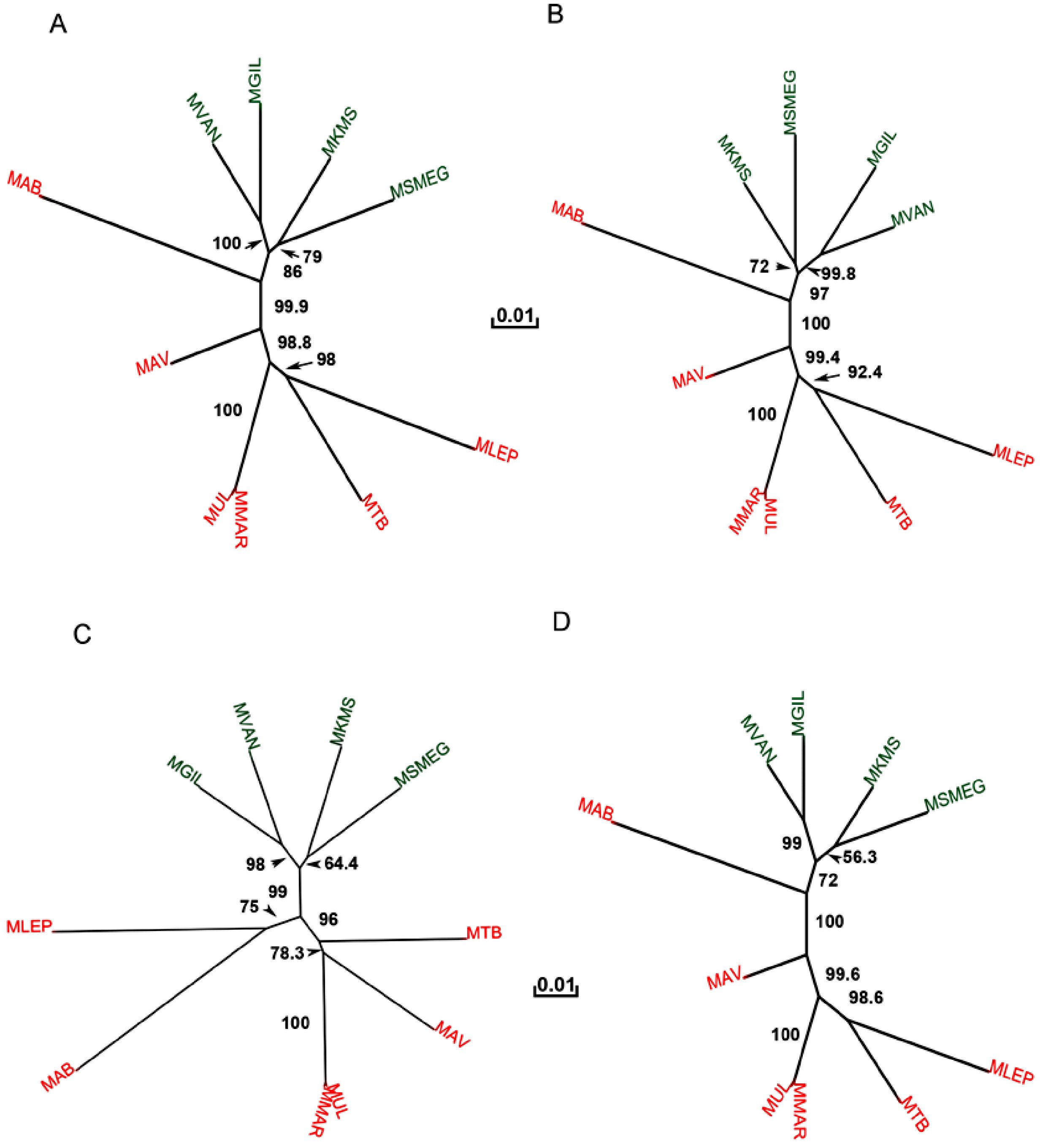

Figure shows the phylogenetic tree for *dnaN* nucleotide sequences drawn by following methods: A) Fitch-Minimum evolution, B) Neighbor-Joining, C) UPGMA, D) Maximum likelihood. The numbers in branches indicate the bootstrap percentage values for 1000 replicates.

Phylogenetic tree based on Gene content (Largest genome)

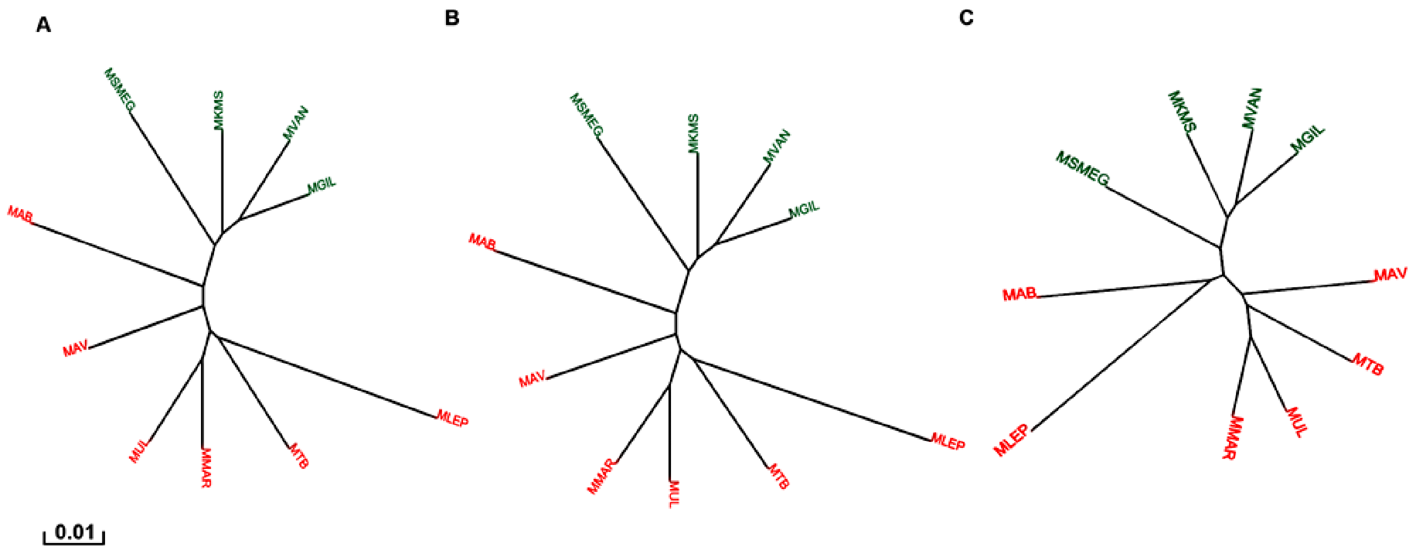

Figure shows the phylogenetic tree for gene content based on % similarity of orthologs computed by largest of two genomes. Trees shown here are drawn by following methods: A) Fitch-Minimum evolution, B) Neighbor-Joining, C) UPGMA

Phylogenetic trees with inclusion of OUTGROUP

Phylogenetic trees were constructed using *Streptomyces coelicolor* (SCO) as an Outgroup. With the inclusion of outgroup, the number of core orthologs reduced from 759 to 275. Trees were constructed using Fitch Margolish method. For tree based on concatenation of protein sequences, neighbour joining method was used. The phylogenetic trees are shown in the figure below. The inclusion of an outgroup did not affect the inference made in the paper.

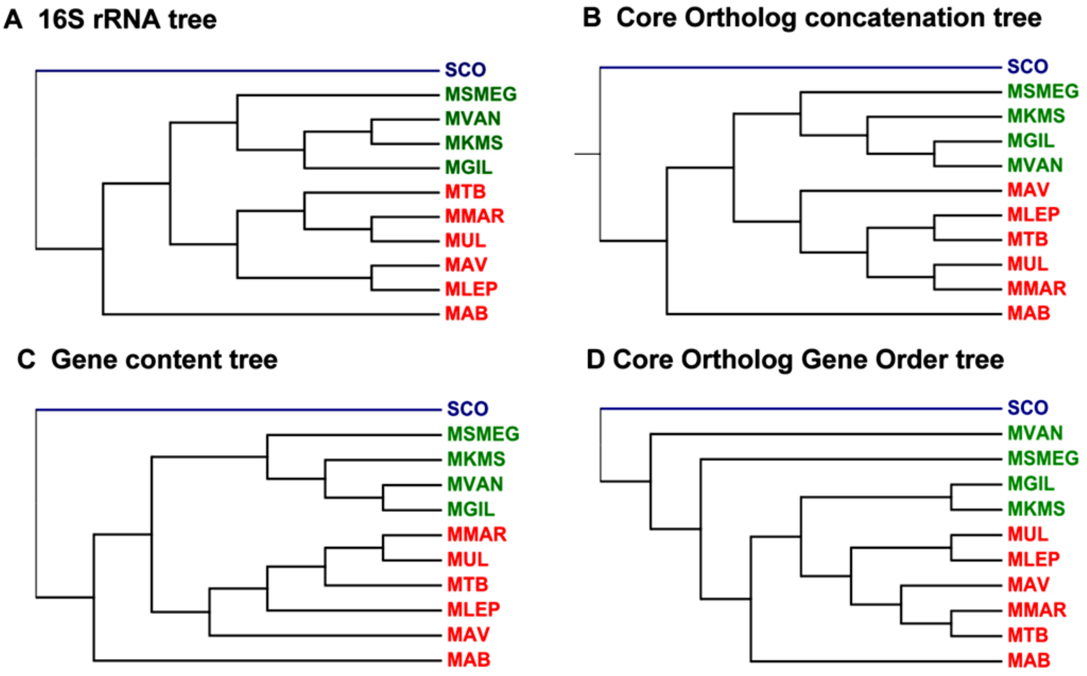

In case of gene order tree (figure D) the distance between SCO and the non-pathogens was same. Therefore, the tree is not well-resolved for the non-pathogens. In contrast, the phylogenetic relationship between the species is well resolved when the full set of 759 core orthologs are considered (see tree reported in main text).
