# Supplementary material for: Comparative Phylogenomics of Pathogenic and Non-Pathogenic Mycobacterium
Source: PLoS One. 2013 Aug 28;8(8):e71248. doi: 10.1371/journal.pone.0071248 (PMC3756022; doi:10.1371/journal.pone.0071248)
Supplement: Figure S5 — BLAST hits for exclusive pathogenic genes in each of the non-pathogens considered in this study. The exclusive genes in M. tuberculosis H37Rv were used as reference for BLAST. (PDF) [file pone.0071248.s005.pdf]

Table-S1: BLAST hits for exclusive pathogenic genes in each of the non-pathogens considered in this study. The exclusive genes in *M. tuberculosis* H37Rv were used as reference for BLAST.

GENE PRODUCT	MTB	MSMEG		MGIL		MKMS		MVAN	
succinate-semialdehyde dehydrogenase	Rv0234c	6452		4860		-		1844	
		46	88	36	90	-	-	43	89
Conserved membrane protein	Rv0364	0750		0237		0512		0688	
		42	96	41	92	42	96	44	92
Orotate phosphoribosyltransferase	Rv0382c	6520		1084		5212		5738	
		38	65	31	86	34	86	32	86
Membrane protein	Rv0451c	0380		-		5264		3834	
		60	99	-	-	50	97	51	99
Transcriptional regulator	Rv1404	5566		0546		5561		0537	
		32	75	25	76	25	76	25	76
Glycosyl transferase	Rv1524	0385		-		3175		-	
		58	99	-	-	62	100	-	-
Hypothetical protein cpsA	Rv3484	0107		4737		1335		1726	
		41	84	38	77	41	77	36	86
Glycosyl transferase	Rv3631	3859		3118		2548		0886	
		25	93	25	93	26	93	30	80
Conserved membrane protein	Rv3632	2455		-		-		-	
		54	24	-	-	-	-	-	-

* The numbers in white row indicate the loci for each organism. For example, the best homolog hit for Rv0234c in *M. smegmatis* was found to be MSMEG_6452. Numbers in orange blocks indicate % identity, while the numbers in green blocks represent % coverage.
