# Supplementary material for: Comparative Phylogenomics of Pathogenic and Non-Pathogenic Mycobacterium
Source: PLoS One. 2013 Aug 28;8(8):e71248. doi: 10.1371/journal.pone.0071248 (PMC3756022; doi:10.1371/journal.pone.0071248)

Multiple sequence alignment of all 9 exclusive genes of pathogens

1) Rv0234c – Succinate semialdehyde dehydrogenase

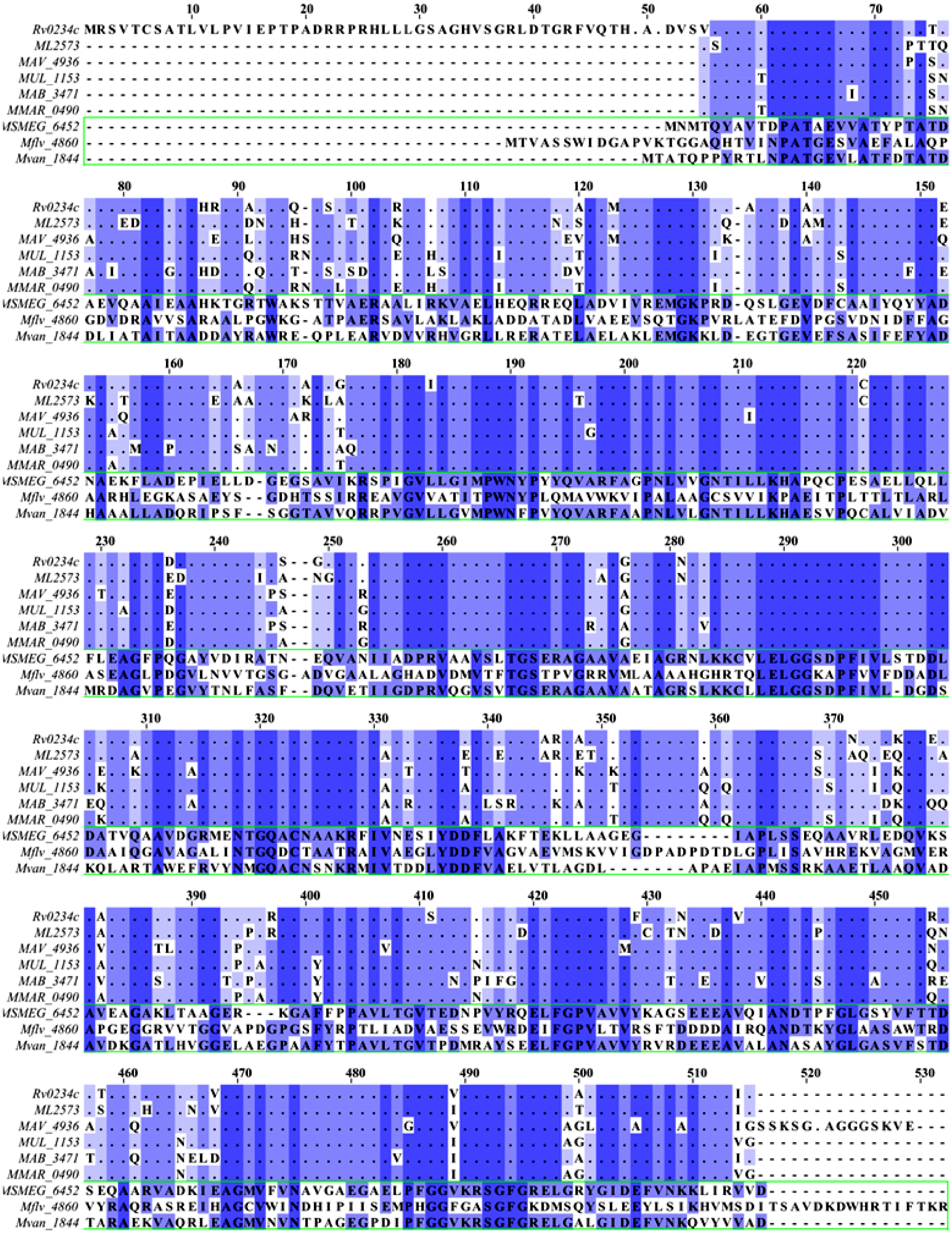

2) Rv0364 – Conserved membrane protein

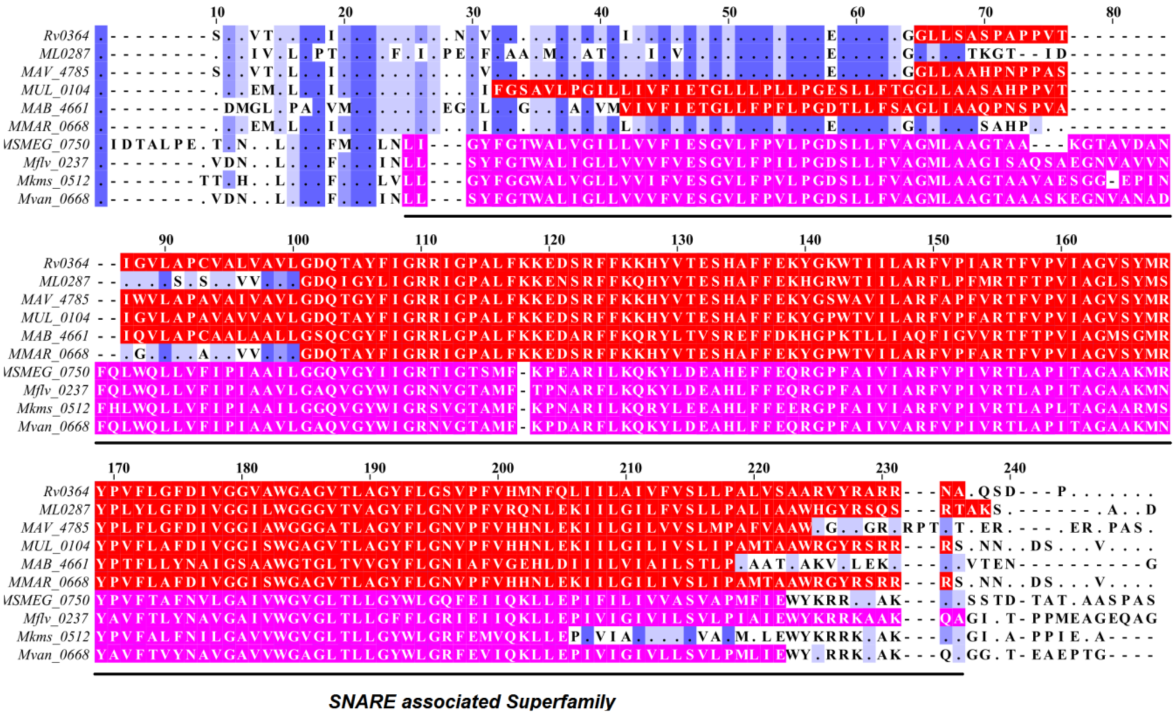

3) Rv0382c – Orotate phosphoribosyltransferase

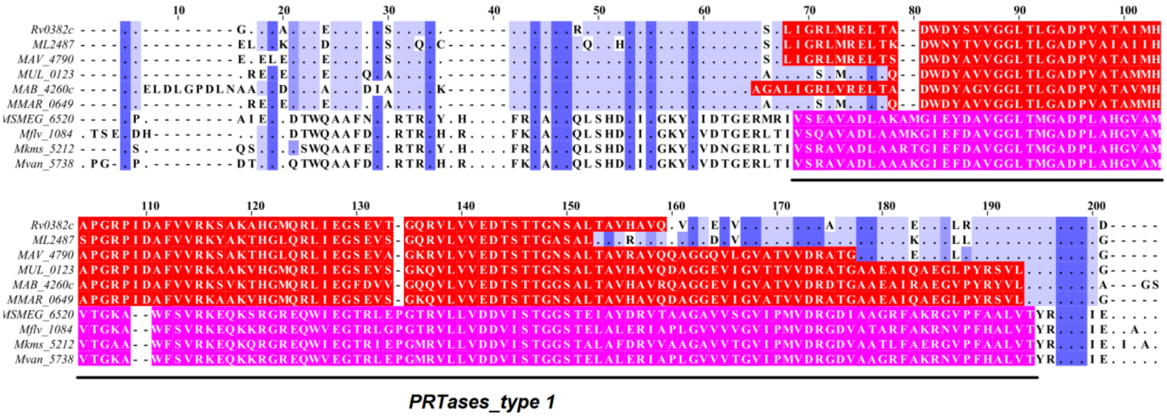

4) Rv0451c – Membrane protein

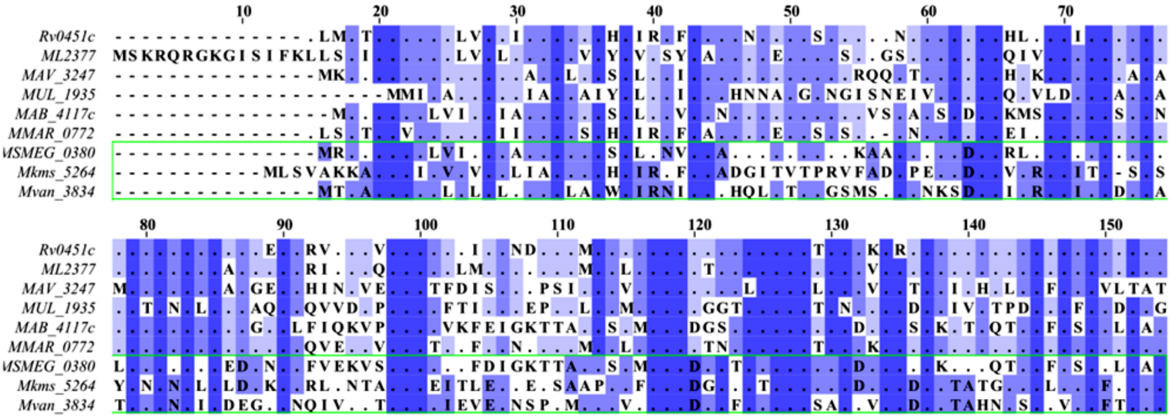

5) Rv1404- Transcriptional regulator

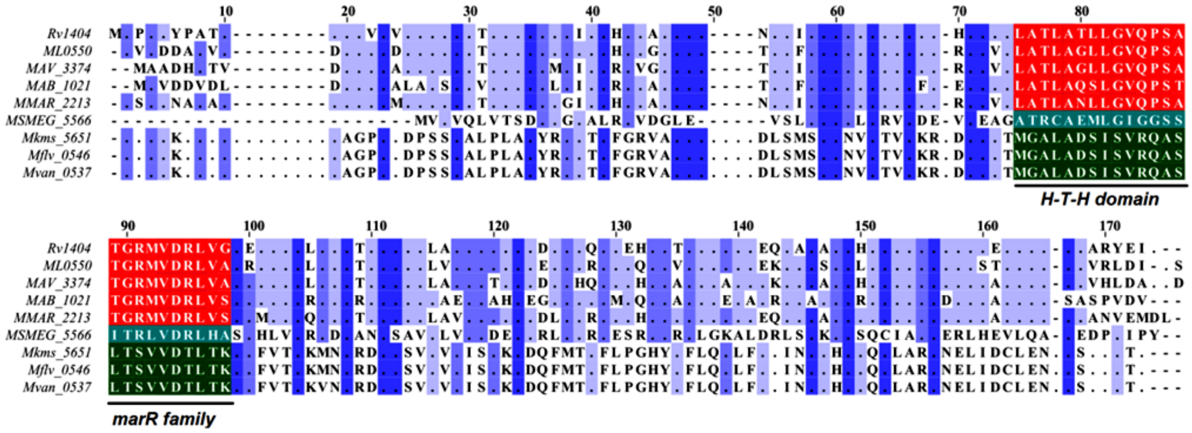

6) Rv1524- Glycosyl transferase

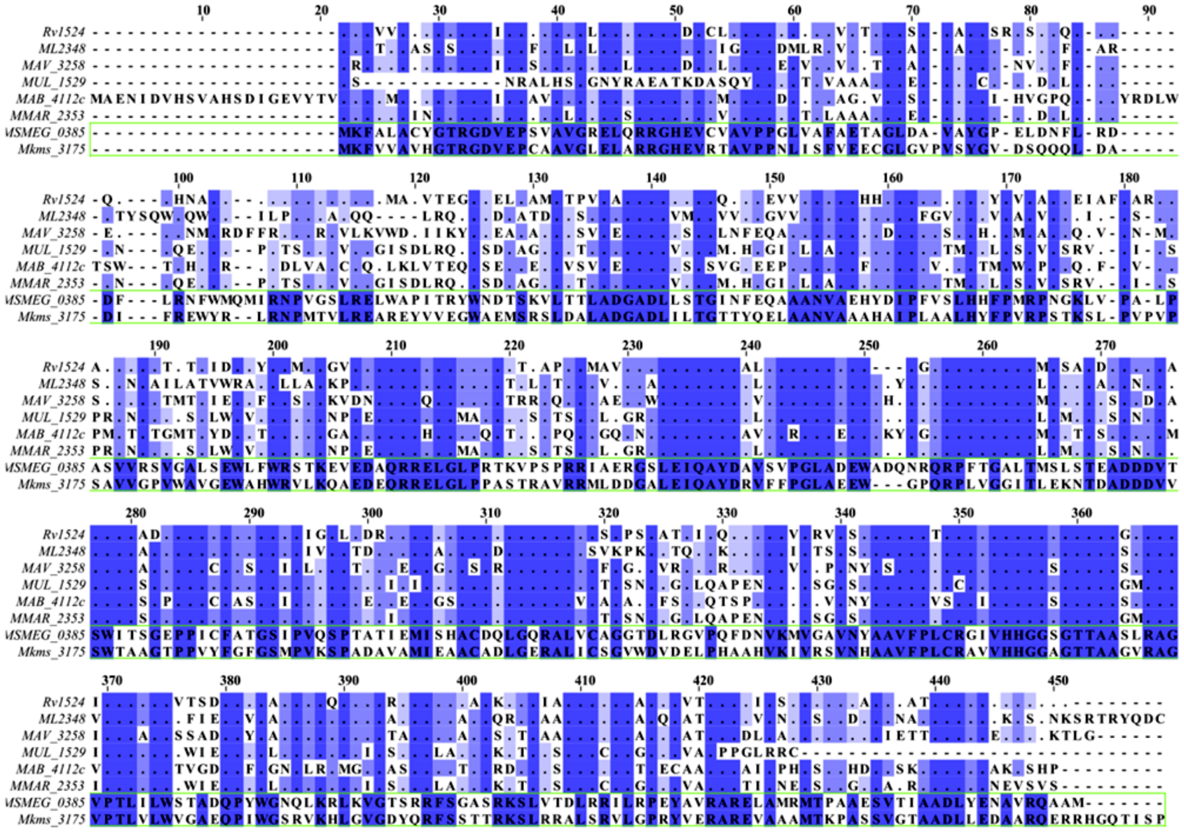

7) Rv3484- Hypothetical protein cpsA

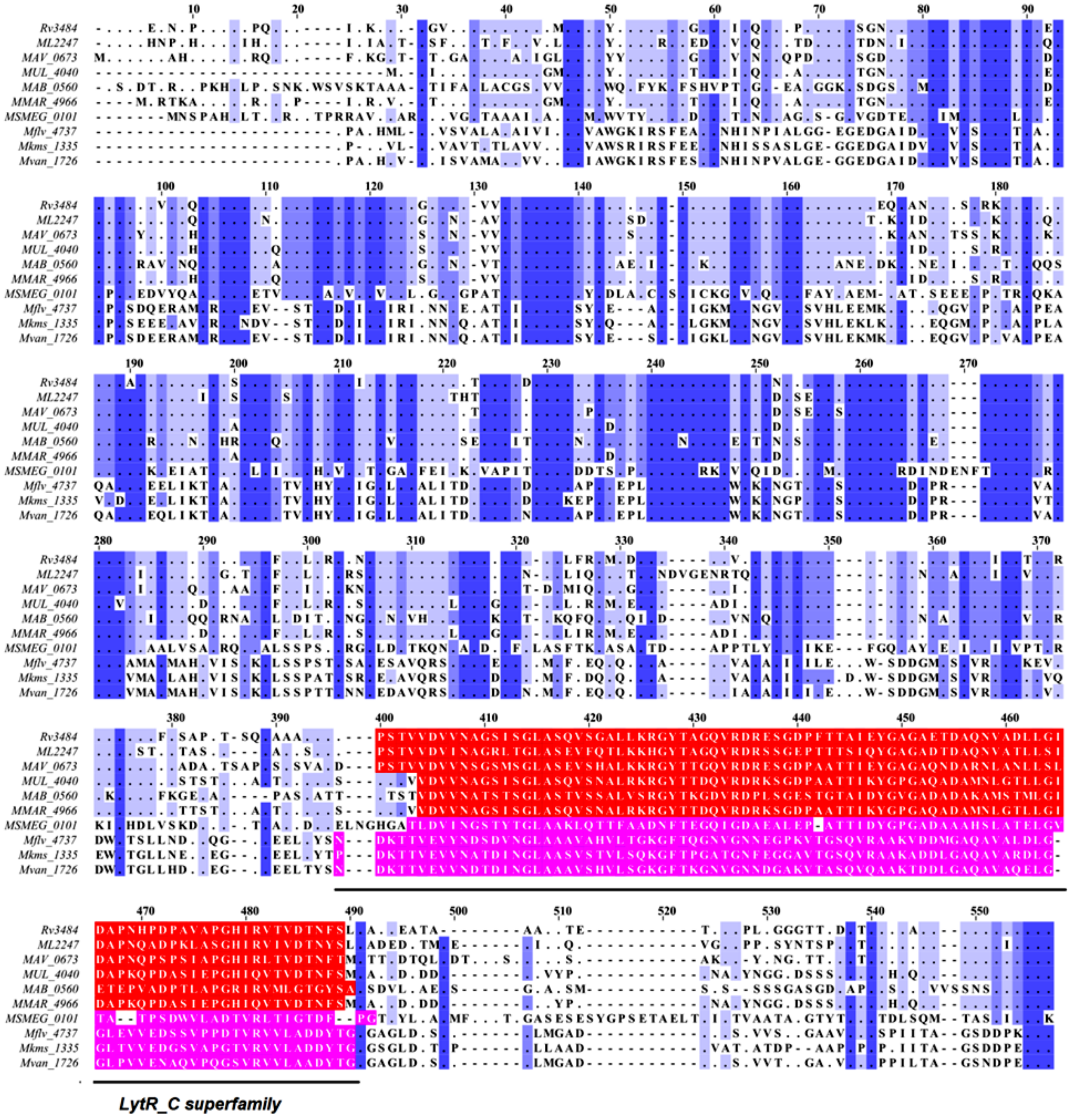

8) Rv3631- Glycosyl transferase

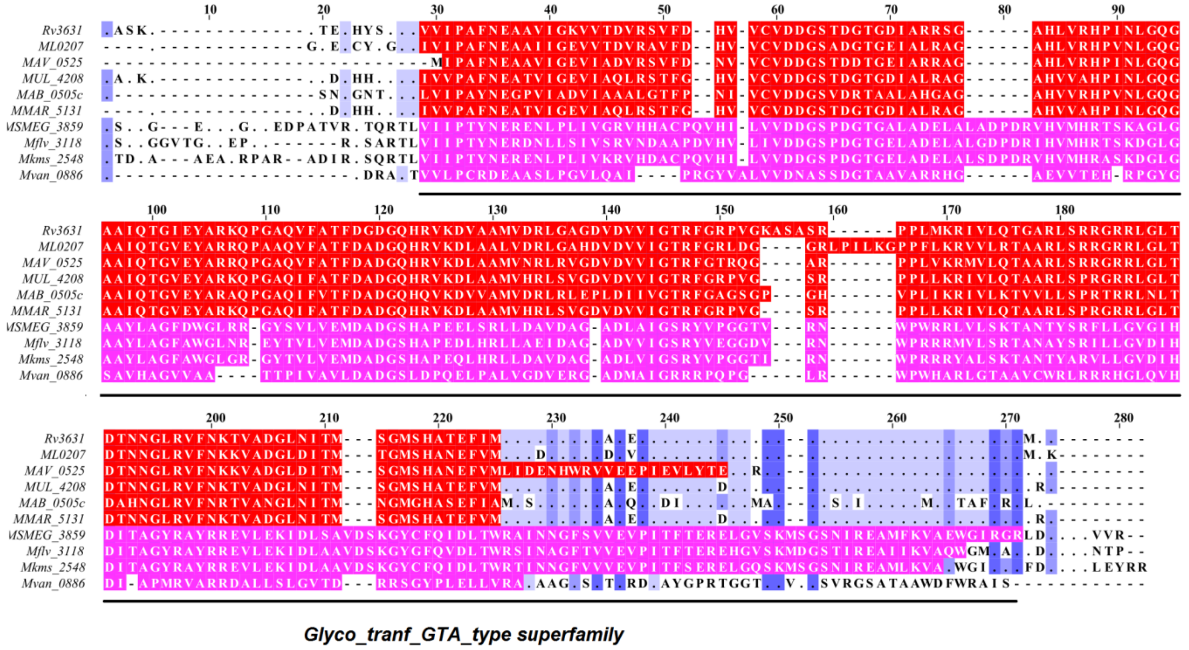

9) Rv3632- Conserved Membrane protein

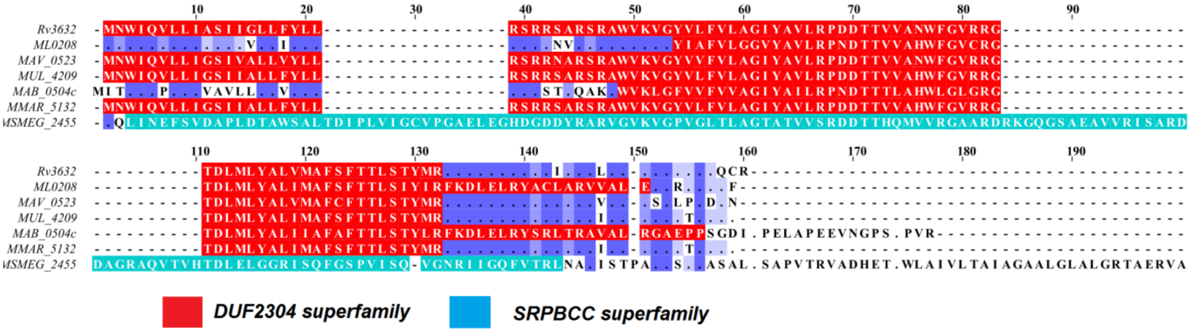

Supplement: Figure S6 — Multiple sequence alignment of all 9 exclusive genes of pathogens. File shows the multiple sequence alignment for each of the 9 exclusive genes present in pathogen for all the ten organisms considered in this study. Conservation of amino acid residue is represented by (.), similarly the gaps are shown by (−). Specially shaded regions indicate the conserved domain of protein families. (PDF) [file pone.0071248.s006.pdf]
