# Supplementary material for: Comparative Phylogenomics of Pathogenic and Non-Pathogenic Mycobacterium
Source: PLoS One. 2013 Aug 28;8(8):e71248. doi: 10.1371/journal.pone.0071248 (PMC3756022; doi:10.1371/journal.pone.0071248)

Plausible origin of genes exclusively present in Mycobacterium pathogens:

We identified 9 genes that were present in all the pathogens, but absent in the non-pathogens. To understand the origin of these genes, protein and nucleotide sequences of each was searched for similar sequences across non-redundant database. Blast hits with 50% identity and at least 60% coverage were considered significant. We observe that for most of the genes the hits belonged to the suborder corynebacterinae while only two genes found hits outside the group. Corynebacteriaceae are closely related to mycobacterinaceae. Both belong to the order actinomycetales. Most of the organisms under this suborder are opportunistic pathogens and soil bacterium. The results are presented in the table below.

Gene	Protein Hits other than mycobacteria	Nucleotide Hits other than mycobacteria
Rv0234c	Cyanobacteria and Proteobacteria	Proteobacteria and archea
Rv0364	Corynebacterinae and Verrucomicrobia	Corynebacterinae and Proteobacteria
Rv0382c	Corynebacterinae	Corynebacterinae
Rv0451c	No hits	No hits
Rv1404	Corynebacterinae	Corynebacterinae
Rv1524	No hits	No hits
Rv3484	Corynebacterinae	Corynebacterinae
Rv3631	Corynebacterinae	Corynebacterinae
Rv3632	Corynebacterinae	Corynebacterinae

BLAST hits outside the mycobacteria or corynebacteria group, suggests that these genes could have been acquired through horizontal gene transfer. However, further investigation is required to prove this. Two genes, Rv0451c and Rv1524 do not have any significant hits other than mycobacterial pathogens. These genes' sequences have probably evolved along with the pathogens, and their specific role in pathogenesis, if any needs further investigation.

Based on the BLAST hits for each gene, a distance tree was constructed as shown in figures below. Species belonging to same genus were condensed and the values of best hit (%

identity and % coverage) are represented as numbers next to the organism's name. (For example: 74-89 means 74% identity and 89% coverage). Identity and coverage for the best hits are also shown.

1) Rv0234c – gabD1: succinate semialdehyde dehydrogenase

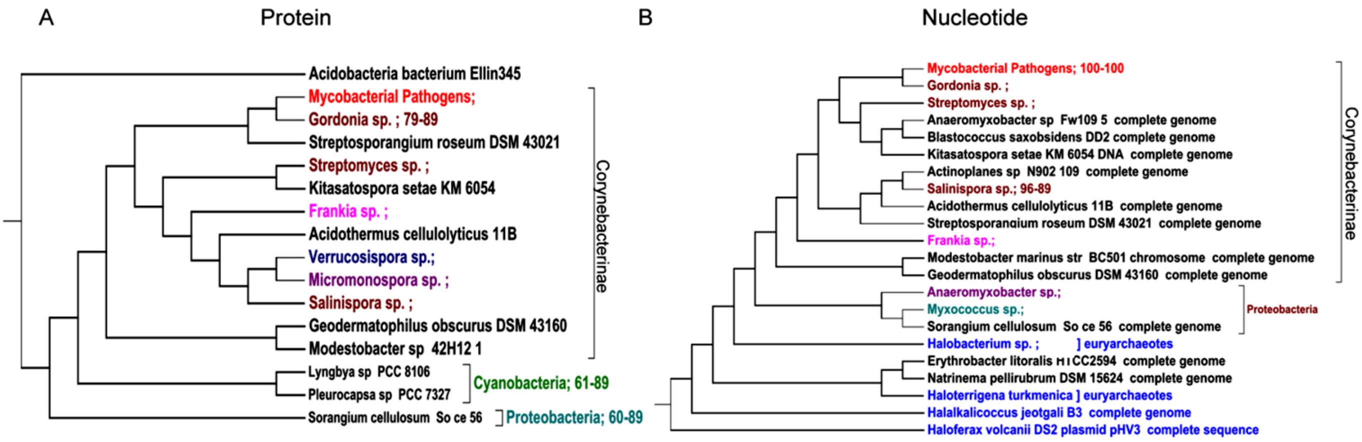

a. Found hit in Pleurocapsa sp. belonging to cyanobacteria at 61% sequence identity with 89% coverage. These are nitrogen fixing bacteria found in marine environment.
b. Found hit in *Sorangium cellulosum* belonging to d-proteobacteria of phylum bacteria at 60% sequence identity with 89% coverage. These are soil-dwelling bacteria and saprophytes.

2) Rv0364 – Conserved membrane protein:

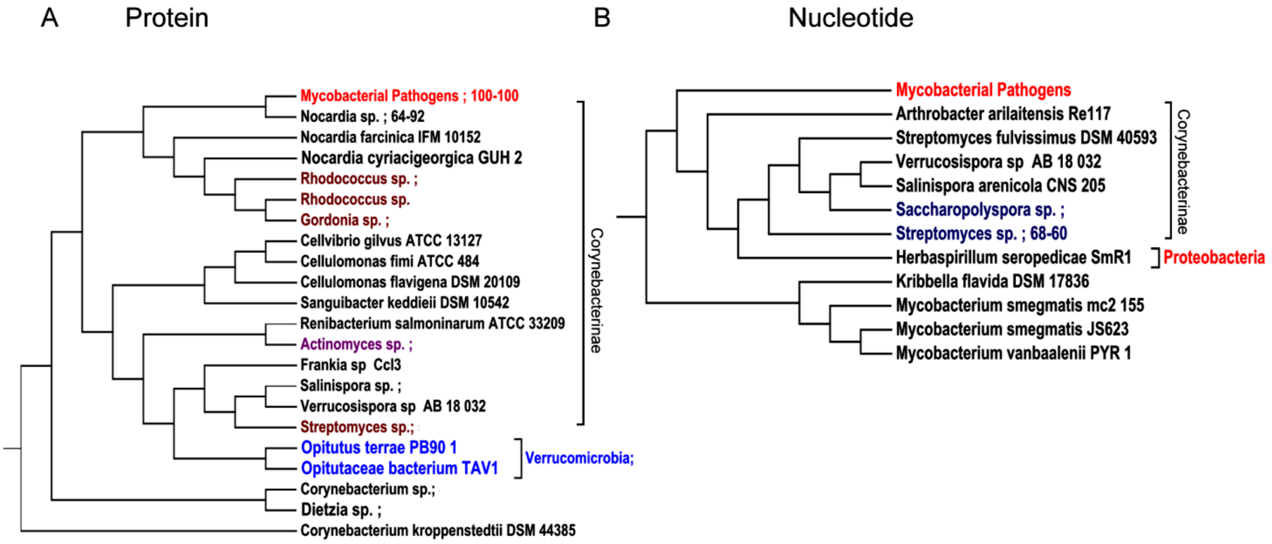

Found hit in *Optitucae* sp. belonging to verrucomicrobia at 48% sequence identity with 82% coverage. Verrucomicrobia belongs to superkingdom bacteria. These organisms are found in soil, fresh water and human faeces. They have been identified in association with eukaryotic hosts including extrusive explosive ectosymbionts of protists and endosymbionts of nematodes residing in their gametes.

3) Rv0382c – Orotate phosphoribosyl transferase (*pyrE*)

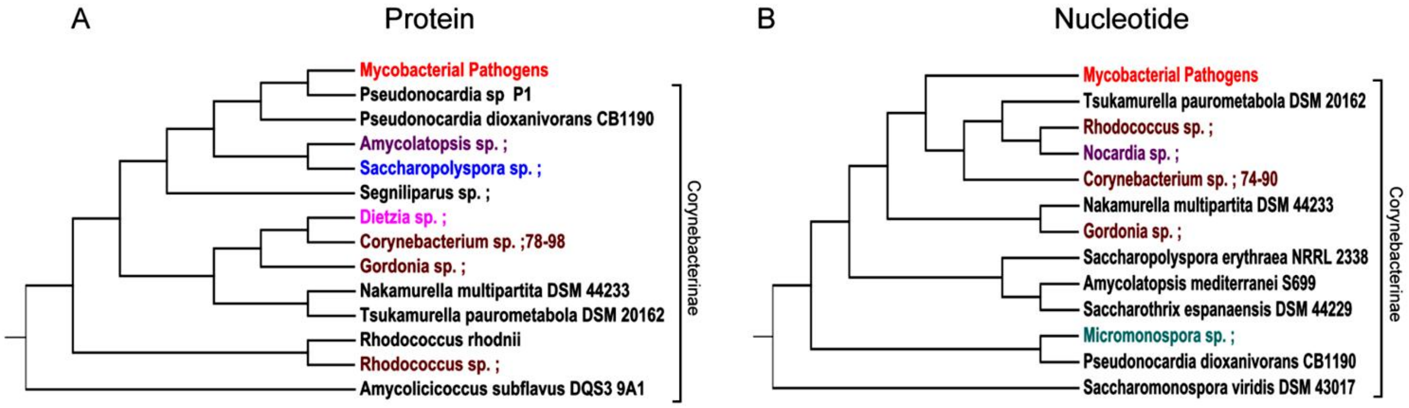

4) Rv1404 – Transcriptional regulator

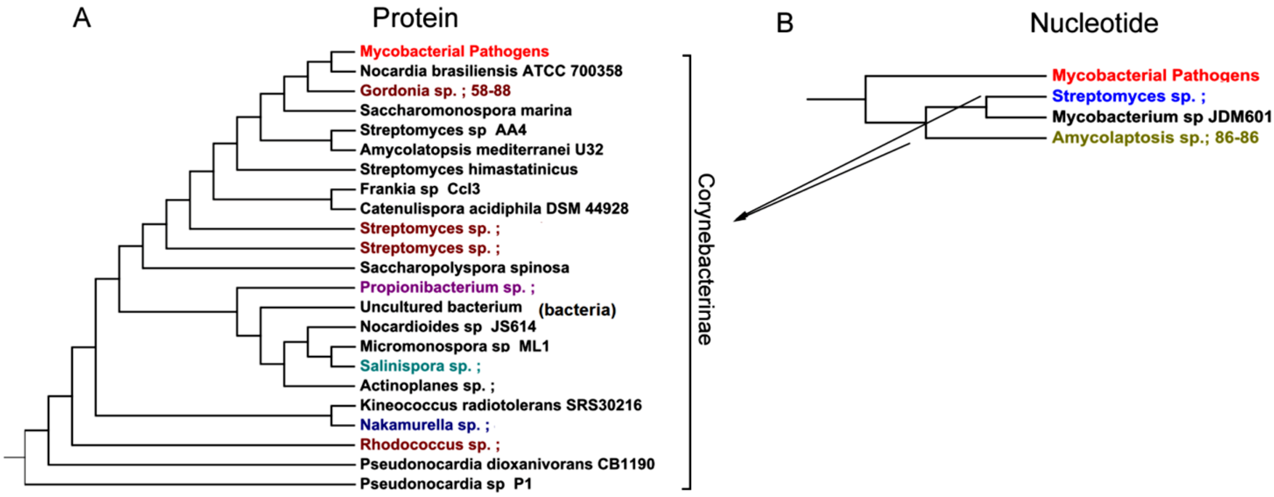

5) Rv3484 – Hypothetical Protein cpsA

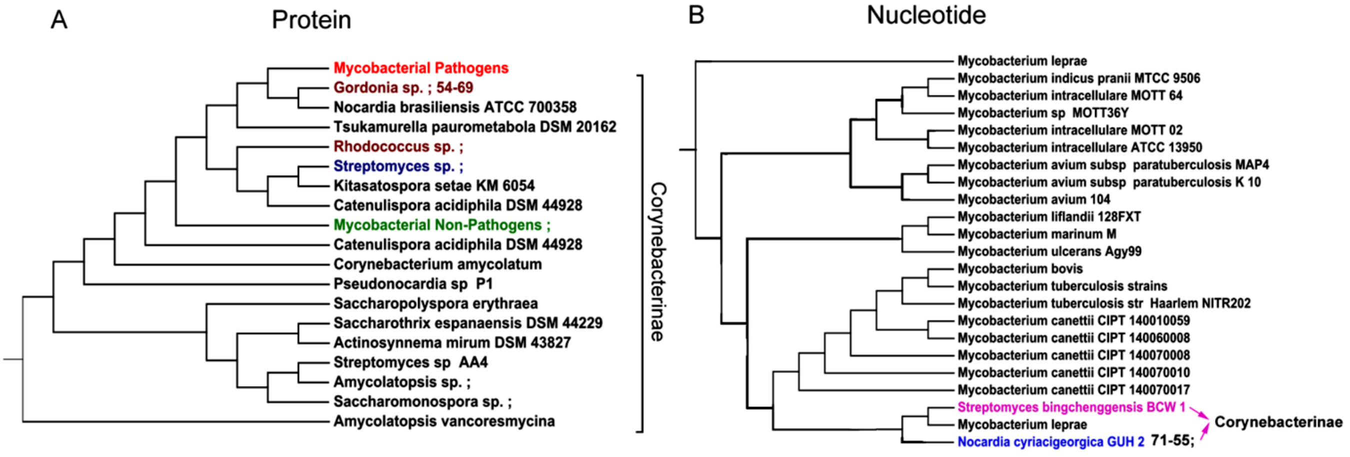

6) Rv1524 – Glycosyl transferase

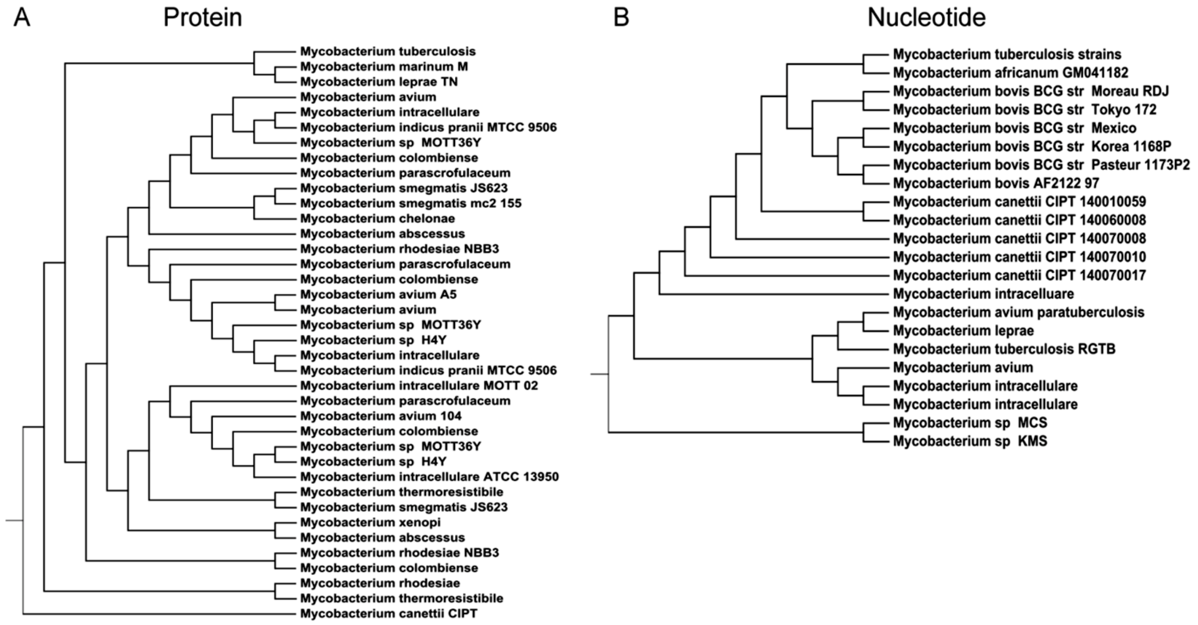

7) Rv3631 – Glycosyl transferase

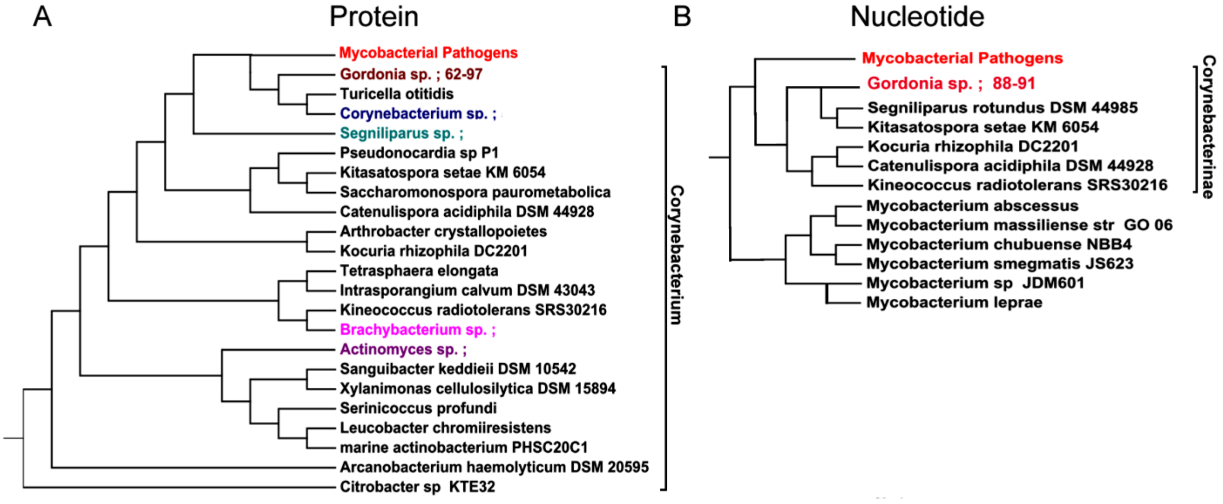

8) Rv3632 – Conserved membrane protein

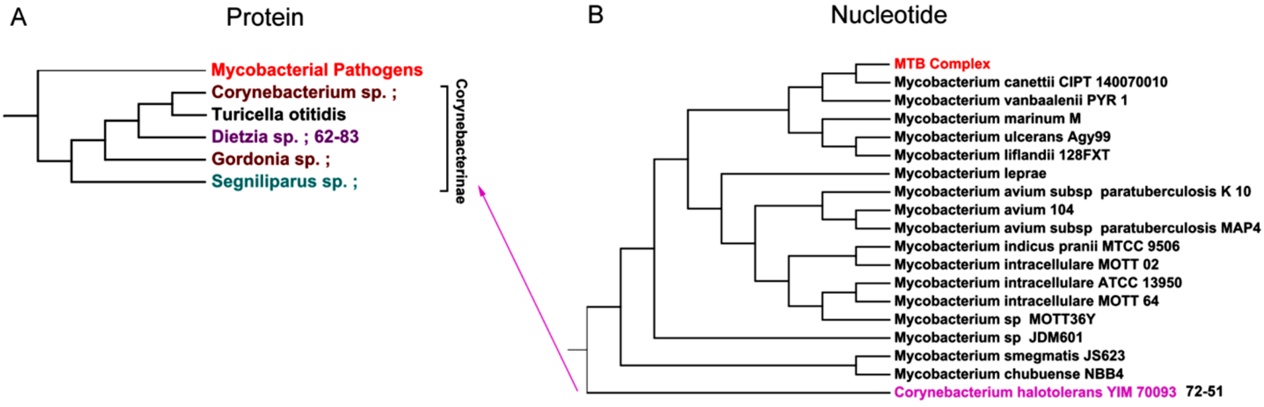

9) Rv0451 - Membrane protein (*mmpS4*)

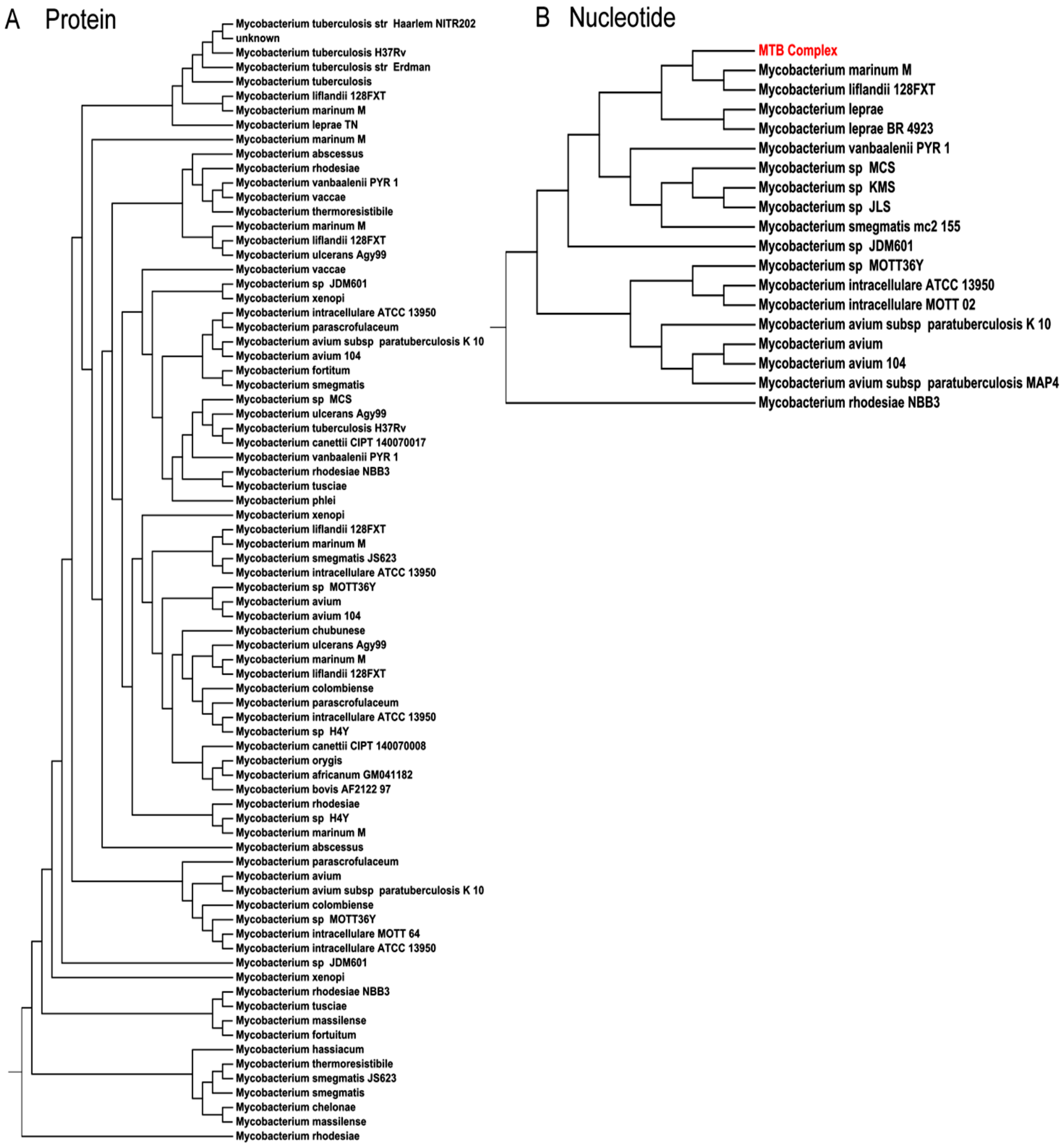

Supplement: Figure S7 — BLAST results to determine plausible origin of genes exclusive to pathogens. (PDF) [file pone.0071248.s007.pdf]
