# Supplementary material for: Comparative Phylogenomics of Pathogenic and Non-Pathogenic Mycobacterium
Source: PLoS One. 2013 Aug 28;8(8):e71248. doi: 10.1371/journal.pone.0071248 (PMC3756022; doi:10.1371/journal.pone.0071248)
Supplement: Figure S8 — Comparison of results based on genomic features with McGuire et. al . (PDF) [file pone.0071248.s008.pdf]

Comparison of general features of mycobacterium based on this work with McGuire *et al.*

The analysis on general features of mycobacterium presented in this paper was compared with that from the paper titled "Comparative analysis of Mycobacterium and related Actinomycetes yields insight into the evolution of Mycobacterium tuberculosis pathogenesis" by McGuire et al. BMC Genomics 2012, 13:120. One of the important differences between the two approaches is the classification method used. While we used the TIGR classification for assigning role category to each gene, McGuire *et al*. use the GO annotation.

Based on the information available at http://www.broadinstitute.org /ftp/pub/seq/msc/ pub/ SYNERGY/gene_sets/metabolic_pathways/pwy_hierarchy.html, we compared our results to that based on the GO classification. The TIGR and GO terms were matched, such as, Fatty acid synthesis and Phospholipid metabolism (FPM) was considered to be equivalent with 'Lipid Biosynthesis'.

The number of genes in many GO classes was found to vary linearly with the genome size, as shown in the figure below.

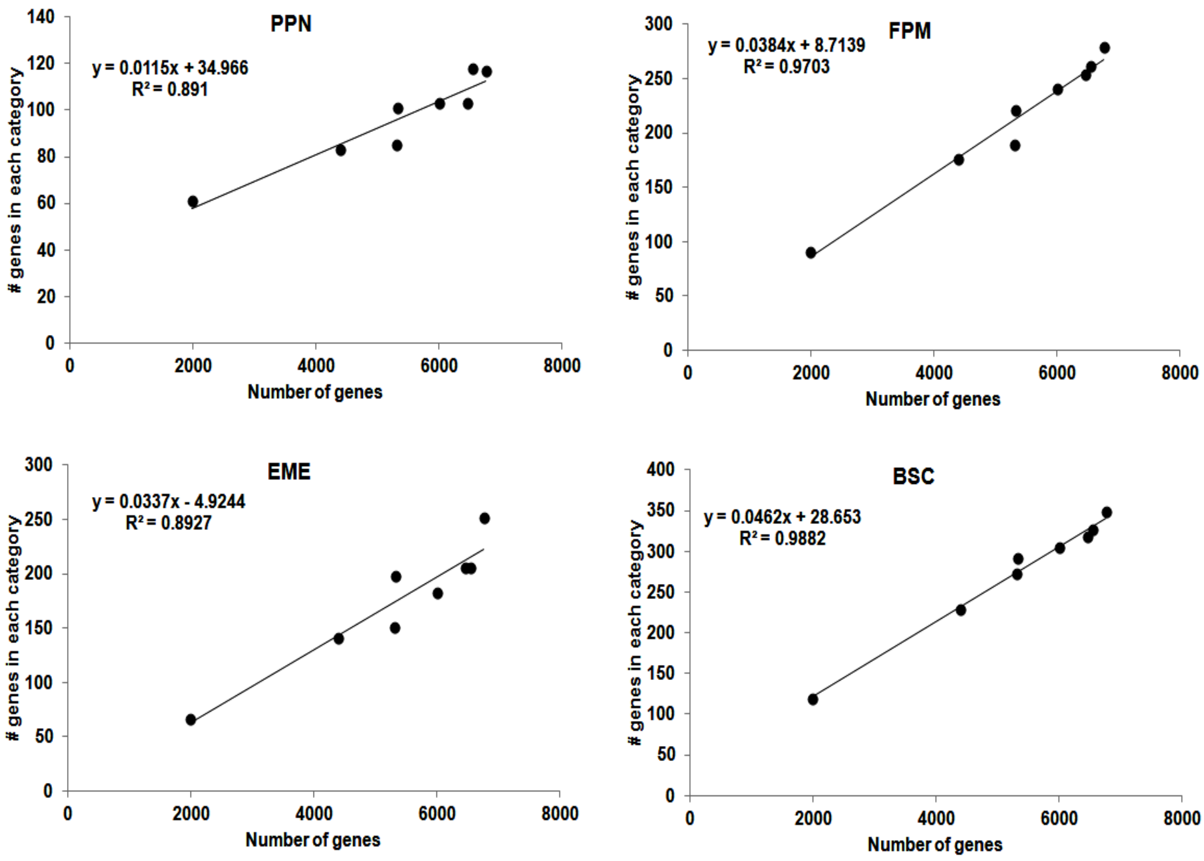

We also found that, the ratio of average number of genes in each category in pathogen to non-pathogens matched between the two datasets as shown in the figure below. Thus, the results are independent of the gene annotation system.

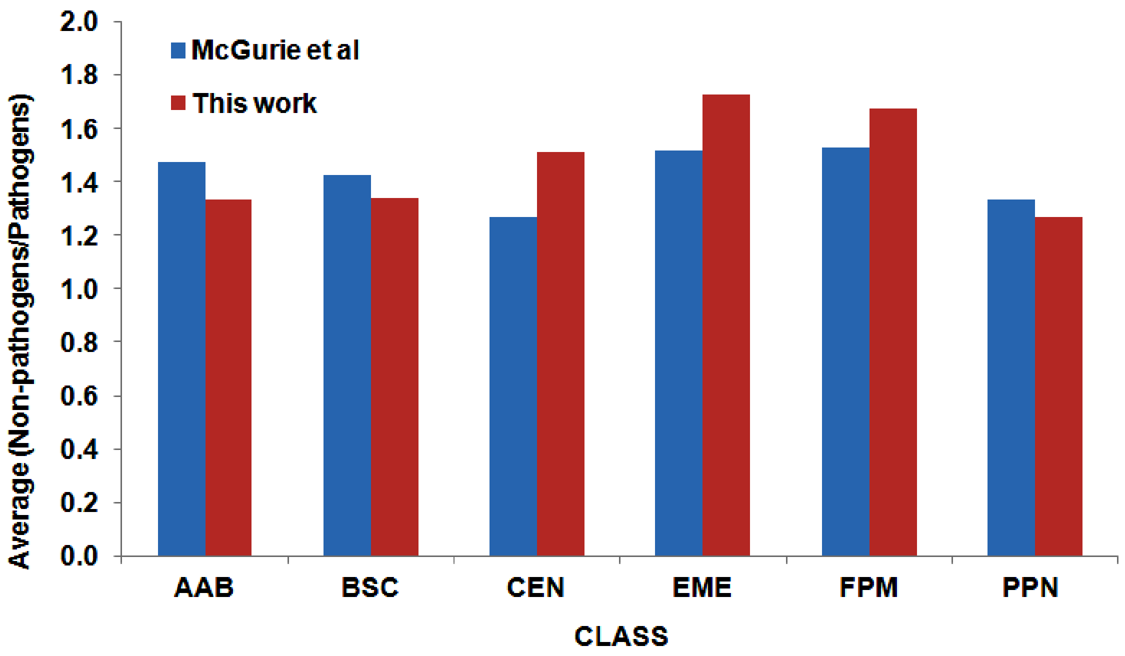
McGurie et al
This work
Average (Non-pathogens/Pathogens)
2.0
1.8
1.6
1.4
1.2
1.0
0.8
0.6
0.4
0.2
0.0
AAB
BSC
CEN
EME
FPM
PPN
CLASS
